# Atopic Dermatitis and Bone Fragility: Osteoimmunological Pathways, Therapeutic Paradoxes, and Risk-Adapted Screening Across the Lifespan

**DOI:** 10.3390/diagnostics16213660

**Published:** 2026-11-05

**Authors:** Ancuța-Ramona Boicea Camen, Mohamed-Zakaria Assani, Rodica Pădureanu, George G. Mitroi, Vlad Pădureanu, Alexandra-Ștefania Stroe-Ionescu, Mihail Virgil Boldeanu, Daniel Cosmin Caragea, Lidia Boldeanu

**Affiliations:** 1 Department of Occupational Medicine, University of Medicine and Pharmacy of Craiova, 200349 Craiova, Romania; ancuta.boicea@umfcv.ro; 2 Doctoral School, University of Medicine and Pharmacy of Craiova, 200349 Craiova, Romania; mohamed.assani@umfcv.ro (M.-Z.A.); alexandra.stroe@umfcv.ro (A.-Ș.S.-I.); 3 Department of Immunology, Faculty of Medicine, University of Medicine and Pharmacy of Craiova, 200349 Craiova, Romania; 4 Department of Internal Medicine, Faculty of Medicine, University of Medicine and Pharmacy of Craiova, 200349 Craiova, Romania; rodica.padureanu@umfcv.ro (R.P.); vlad.padureanu@umfcv.ro (V.P.); 5 Department of Dermatology, University of Medicine and Pharmacy of Craiova, 200349 Craiova, Romania; mitroi.george@yahoo.com; 6 Research Center of Periodontal-Systemic Interactions, University of Medicine and Pharmacy of Craiova, 200349 Craiova, Romania; 7 Department of Nephrology, Faculty of Medicine, University of Medicine and Pharmacy of Craiova, 200349 Craiova, Romania; 8 Department of Microbiology, Faculty of Medicine, University of Medicine and Pharmacy of Craiova, 200349 Craiova, Romania; lidia.boldeanu@umfcv.ro

**Keywords:** atopic dermatitis, osteoporosis, bone fragility, fractures, bone mineral density, osteoimmunology, RANKL, dupilumab, glucocorticoids, DXA

## Abstract

Atopic dermatitis (AD) has been linked to osteoporosis and fractures, but the strength, mechanisms, treatment implications, and clinical significance of this relationship remain uncertain. This review critically assesses epidemiological and osteoimmunological evidence connecting AD with skeletal fragility and proposes a lifespan-based, risk-adapted screening framework. A recent cohort-based meta-analysis found associations between AD and osteoporosis (odds ratio [OR], 1.56) and any fracture (OR, 1.08), although heterogeneity was substantial. Risk appeared higher with severe or long-standing disease and reflected interactions among inflammatory, therapeutic, nutritional, behavioral, and age-related factors. Systemic glucocorticoids were the clearest modifiable treatment-related risk, but did not fully account for the association. The RANK–RANKL–osteoprotegerin system and IL-4, IL-13, IL-31, and IL-33 offer biological plausibility, although their skeletal effects are context-dependent and supported primarily by indirect or preclinical evidence. Available data do not show that dupilumab causes osteoporosis. Emerging pediatric findings suggest improvements in growth and bone-related biomarkers, but provide no evidence of fracture prevention. AD alone does not warrant universal DXA screening. Assessment should be individualized based on age, history of fragility fractures, disease severity and duration, cumulative systemic glucocorticoid exposure, nutritional status, physical activity, muscle function, and falls. Prospective studies combining standardized AD phenotyping with longitudinal imaging, biomarkers, and adjudicated fractures are needed.

## 1. Introduction

Atopic dermatitis (AD) is a chronic, relapsing inflammatory skin disease that can begin in infancy, persist into adulthood, or first present later in life. Its clinical expression reflects a dynamic interaction among epidermal-barrier dysfunction, microbial dysbiosis, neuroimmune signaling, and immune polarization, with type 2 pathways predominating in many—but not all—phenotypes [1,2,3,4,5]. This biological heterogeneity, together with a substantial burden of pruritus, sleep loss, psychosocial impairment, and treatment exposure, has shifted the conceptual model of AD from an isolated dermatosis toward a systemic, life-course disorder with clinically relevant extracutaneous comorbidities [6,7,8,9,10].

Bone fragility has emerged as one of the least resolved components of this expanded comorbidity spectrum [11,12,13]. The association is clinically important because AD frequently develops before attainment of peak bone mass, may persist for decades of skeletal remodeling, and often coexists with factors that can impair bone health, including systemic glucocorticoid exposure, reduced physical activity, nutritional restriction, vitamin D insufficiency, and chronic sleep disruption. Conversely, osteoporosis and fracture are age-dependent outcomes whose interpretation requires attention to sex, menopausal status, growth, skeletal maturation, and the distinction between bone mineral density (BMD) and bone quality. A single, uniform explanation for the AD–bone relationship is therefore unlikely.

The epidemiologic signal is nevertheless increasingly consistent. Systematic reviews and meta-analyses published since 2020 have linked AD with lower BMD, osteopenia or osteoporosis, and fractures, while population-based studies have extended these observations to children, young adults, and older adults [12,13,14,15,16,17,18,19,20,21,22,23,24,25,26,27,28,29,30,31,32]. The most recent meta-analysis restricted to cohort studies reported a pooled odds ratio of 1.56 for osteoporosis and 1.08 for any fracture in people with AD, although between-study heterogeneity remained substantial [31]. Individual cohorts also suggest that risk is not evenly distributed: greater disease severity, longer duration, older age, and systemic corticosteroid use repeatedly identify higher-risk subgroups [14,15,16,17,18,24,25,26,30]. At the same time, variation in case definitions, exposure ascertainment, fracture coding, BMD testing, disease severity, and adjustment for lifestyle and medication creates uncertainty about the magnitude—and, more importantly, the causality—of the association.

A plausible biological bridge is provided by osteoimmunology. Bone remodeling depends on coordinated communication among osteoblast-lineage cells, osteocytes, osteoclasts, and immune cells, with the receptor activator of nuclear factor kappa-B (RANK)–RANK ligand (RANKL)–osteoprotegerin (OPG) axis serving as a central final pathway of osteoclast differentiation and bone resorption [33,34,35,36,37,38,39,40,41,42,43]. In older women with AD, the circulating RANKL/OPG ratio has been reported to correlate with disease severity, providing a direct, although cross-sectional, link between cutaneous inflammation and a skeletal remodeling signal [14,22]. Yet the cytokine biology is not unidirectional. IL-4 and IL-13 can suppress osteoclastogenesis, promote anti-inflammatory macrophage phenotypes, or increase OPG in several experimental systems [38,39,42,44], whereas excessive or context-dependent type 2 inflammation can produce different effects, including enhanced osteoclastogenesis in allergic inflammation [45,46,47]. IL-31 and IL-33 add further complexity because their effects vary across target cells, tissues, disease models, and inflammatory milieus [34,36,40,41]. These findings support biological plausibility, but most derive from non-AD or preclinical models and should not be interpreted as proof of a direct cytokine-to-fracture pathway in patients.

Non-inflammatory mediators may be equally important. Oral glucocorticoids have well-established adverse effects on osteoblast survival, bone formation, calcium balance, muscle function, and fracture risk, and contemporary osteoporosis guidance treats prolonged exposure as a major secondary cause requiring active risk assessment [48,49,50]. In AD cohorts, oral corticosteroid exposure partly modifies, but does not consistently abolish, the observed fracture association [17,25,26]. Evidence regarding topical corticosteroids is more reassuring but remains limited by confounding factors such as disease severity, cumulative dose, body-surface area, and potency [19,21]. Nutritional restriction and vitamin D status introduce a second layer of ambiguity: recent meta-analyses of vitamin D supplementation in AD reach differing conclusions regarding improvements in skin severity, while evidence from dietary interventions remains heterogeneous [51,52,53,54]. Reduced exercise capacity, lower weekly activity, maladaptive lifestyle behavior, and sleep disturbance may further influence muscle–bone loading, falls, and overall skeletal health [55,56,57]. Current guidelines favor targeted systemic therapies, including biologics targeting IL-4/IL-13 signaling and Janus kinase (JAK) inhibitors, over prolonged systemic glucocorticoid use in patients with moderate-to-severe AD [58,59,60,61]. From a skeletal perspective, these agents may offer indirect benefits by reducing glucocorticoid exposure and improving inflammatory control, physical activity, sleep, and nutritional status. However, their direct effects on bone remodeling remain incompletely understood. IL-4 and IL-13 can inhibit osteoclast differentiation in experimental models, raising theoretical questions about the consequences of blocking these pathways [38,39,42,44]. Emerging pediatric evidence suggests that dupilumab treatment may improve linear growth and bone formation-related biomarkers, although effects on BMD and fracture risk have not been established [59]. Selective IL-13 inhibitors and JAK inhibitors may also influence immune–bone interactions, but longitudinal clinical evidence evaluating their skeletal effects in AD remains limited. Thus, targeted therapies may reduce glucocorticoid-associated skeletal risk without necessarily exerting direct osteoprotective effects, as discussed further in Section 6.

Targeted therapy creates a therapeutic paradox. Effective control of AD may improve sleep, mobility, nutrition, and quality of life and may reduce reliance on systemic glucocorticoids, all of which could favor bone health. Conversely, IL-4/IL-13 blockade disrupts cytokine signaling with context-dependent effects on skeletal remodeling, rendering simple mechanistic predictions unreliable [38,39,42,46,47]. Long-term dupilumab studies and pharmacovigilance analyses provide broad safety reassurance, but they were not designed to detect changes in BMD, bone microarchitecture, or incident fragility fractures [58,59,60,61,62,63,64]. Notably, a 2026 post hoc pediatric analysis associated dupilumab treatment with improved linear growth and favorable changes in bone biomarkers [59]. This finding is clinically encouraging, but it does not establish an antifracture effect and cannot be extrapolated to adult or older populations. Within the review window, no prospective adult study was identified that combined baseline and follow-up DXA, standardized bone turnover markers, fracture adjudication, and an appropriate untreated or active comparator.

The diagnostic question is therefore not whether every patient with AD should undergo DXA, but which patients are sufficiently enriched for skeletal risk to justify evaluation. General osteoporosis recommendations prioritize age, sex, menopausal status, prior fragility fracture, and validated clinical risk assessment, whereas glucocorticoid-associated osteoporosis follows a distinct pathway [48,50,65,66,67,68,69]. DXA interpretation must also be age-appropriate: Z-scores, rather than T-scores, are central in children, premenopausal women, and younger men, and low BMD alone does not carry the same diagnostic meaning across these groups [67,68]. Current dermatology guidelines acknowledge bone-related comorbidity but do not yet provide a validated AD-specific screening algorithm [7,9,10,60,61].

Against this background, the present review critically synthesizes literature published from 1 January 2020 through 31 July 2026. Its objectives are to define the strength and limitations of the epidemiologic association between AD, low BMD, osteoporosis, and fracture; distinguish disease-related inflammation from lifestyle-, nutritional-, and treatment-mediated risk; examine the context-dependent skeletal implications of type 2 cytokines and targeted therapy; and develop an age-sensitive, risk-adapted framework for clinical assessment and future research. By integrating population evidence with osteoimmunology and therapeutic data, the review aims to move the field from a broad statement of association toward clinically testable risk stratification.

## 2. Review Methodology

### 2.1. Review Design and Objectives

This study was designed as a critical narrative review supported by a structured literature search. The methodology was selected to accommodate the heterogeneity of the available evidence, which included population-based cohorts, cross-sectional studies, clinical trials, systematic reviews, meta-analyses, mechanistic investigations, pharmacovigilance analyses, clinical guidelines, and translational studies. Because the included publications differed substantially in population characteristics, skeletal outcomes, AD definitions, follow-up duration, and analytical methods, no additional quantitative pooling was performed.

The review was structured around five complementary evidence domains: (1) epidemiologic associations between AD and low BMD, osteoporosis, or fracture; (2) osteoimmunological mechanisms potentially linking cutaneous inflammation with bone remodeling; (3) treatment- and lifestyle-related modifiers of skeletal risk; (4) the potential skeletal implications of targeted therapy; and (5) age-sensitive diagnostic assessment and risk-adapted screening.

### 2.2. Literature Search Strategy

A structured search of PubMed/MEDLINE was conducted for articles first published between 1 January 2020 and 31 July 2026. Crossref records, journal websites, and publisher platforms were subsequently consulted to verify bibliographic metadata, publication dates, article status, pagination, and DOI accuracy. The reference lists of relevant systematic reviews, meta-analyses, guidelines, and key original studies were also manually screened to identify additional eligible publications.

The search strategy combined controlled vocabulary, when available, with free-text terms related to AD and skeletal health. The principal search concepts included: “atopic dermatitis,” “atopic eczema,” and “eczema”; “osteoporosis,” “osteopenia,” “bone mineral density,” “bone fragility,” “fracture,” “fragility fracture,” “bone turnover,” and “bone remodeling”; “osteoimmunology,” “osteoclast,” “osteoblast,” “osteocyte,” “RANK,” “RANKL,” and “osteoprotegerin”; “interleukin-4 (IL-4),” “IL-13,” “IL-31,” “IL-33,” and “type 2 inflammation”; “glucocorticoids,” “corticosteroids,” “vitamin D,” “nutrition,” “dietary restriction,” “physical activity,” “exercise,” and “sleep”; “dupilumab,” “biologic therapy,” “targeted therapy,” “JAK inhibitor,” and “systemic therapy”; and “dual-energy X-ray absorptiometry,” “DXA,” “T-score,” “Z-score,” “screening,” and “fracture risk assessment.”

Search terms were combined using the Boolean operators “AND” and “OR.” Broader searches were initially used to identify epidemiologic and clinical evidence, followed by targeted searches addressing specific mechanisms, treatments, risk modifiers, and diagnostic considerations.

### 2.3. Eligibility Criteria and Study Selection

Publications were considered eligible when they met at least one of the following criteria:

- Evaluated BMD, osteopenia, osteoporosis, fracture incidence, fracture risk, bone turnover markers, or skeletal growth in patients with AD;
- Investigated clinical factors that could modify skeletal risk in AD, including disease severity, disease duration, age, systemic or topical corticosteroid exposure, physical activity, nutrition, vitamin D status, or sleep disturbance;
- Examined immune pathways relevant to both AD and bone remodeling, particularly the RANK–RANKL–OPG system and the effects of IL-4, IL-13, IL-31, or IL-33;
- Assessed the long-term efficacy or safety of targeted AD therapy and reported outcomes potentially relevant to skeletal health, growth, or bone biomarkers;
- Provided current guidance on AD management, osteoporosis prevention, DXA interpretation, or fracture-risk assessment; or
- Contributed directly to the development of an age-sensitive and risk-adapted approach to skeletal evaluation.

Priority was given to original cohort studies, population-based investigations, controlled clinical studies, systematic reviews, meta-analyses, and evidence-based clinical guidelines. Mechanistic studies performed in non-AD inflammatory conditions, cell cultures, or animal models were included only when they addressed pathways directly relevant to the proposed AD–bone relationship. Such evidence was interpreted as biologically informative rather than as direct clinical proof.

Articles were excluded if they were first published before 1 January 2020, did not provide information relevant to AD or skeletal biology, represented duplicate reports without additional data, or lacked sufficient methodological or bibliographic information for evaluation. Two frequently cited papers assigned to 2020 print issues but first published online in 2019 were excluded to preserve the predefined first-publication window.

Following removal of duplicate or clearly irrelevant records, titles and abstracts were screened for relevance. Potentially eligible publications were then assessed through their abstracts, full texts when available, and verified journal records.

### 2.4. Evidence Extraction and Thematic Synthesis

For each included publication, the following information was considered where applicable: study design, country or data source, sample size, age group, definition and severity of AD, duration of follow-up, corticosteroid or targeted-therapy exposure, skeletal outcome, method of BMD assessment, fracture definition, covariates included in adjusted analyses, principal effect estimates, and major limitations.

The evidence was organized into six predefined thematic categories:

- AD context, pathogenesis, and comorbidity framing;
- Direct clinical and epidemiologic evidence concerning AD and bone health;
- Osteoimmunology and type 2 cytokine mechanisms;
- Treatment-, nutritional-, behavioral-, and lifestyle-related risk modifiers;
- Targeted therapy and potential skeletal implications; and
- Diagnostic assessment and risk-adapted screening.

Evidence synthesis emphasized consistency across study designs, temporality, dose- or severity-related patterns, biological plausibility, and the extent to which associations persisted after adjustment for age, sex, corticosteroid exposure, comorbidities, and lifestyle factors. Particular attention was given to distinguishing association from causation and to separating disease-related inflammation from treatment exposure and behavioral or nutritional mediators.

Clinical and preclinical evidence was not assigned equivalent evidentiary weight. Population-based longitudinal studies and meta-analyses were prioritized when evaluating osteoporosis and fracture risk, whereas experimental studies were used primarily to assess mechanistic plausibility. Similarly, pharmacovigilance signals and uncontrolled long-term extension studies were interpreted with caution because they were not designed to detect changes in BMD or fracture incidence.

### 2.5. Methodological Limitations

This review was narrative rather than systematic and was not prospectively registered. Formal risk-of-bias instruments were not uniformly applied due to the considerable heterogeneity of the included evidence, which ranged from experimental studies and clinical guidelines to retrospective database analyses and prospective cohort studies. The search was centered on PubMed/MEDLINE and was supplemented by manual citation screening and publisher-level verification; therefore, relevant publications indexed exclusively in other databases may have been missed.

Additional limitations arose from the underlying literature. Definitions of AD, osteoporosis, osteopenia, and fracture varied across studies, and disease severity was frequently inferred from treatment patterns or diagnostic codes rather than standardized clinical scores. BMD measurements were not universally available, corticosteroid exposure was inconsistently quantified, and residual confounding by physical activity, nutrition, smoking, body composition, sleep disturbance, and comorbid disease remained possible. These limitations were explicitly considered when interpreting the strength, clinical relevance, and causal implications of the available evidence.

This review was conducted as a narrative review and therefore did not follow the Preferred Reporting Items for Systematic Reviews and Meta-Analyses (PRISMA) statement. No formal meta-analysis, quantitative evidence grading, or systematic risk-of-bias assessment of the included studies was performed.

During manuscript preparation, generative artificial intelligence tools, including ChatGPT (GPT-5.5, OpenAI) and Claude (Claude Sonnet 5, Anthropic), were used to assist with the organization and structuring of the review, synthesis and presentation of relevant literature, language editing and refinement, verification and formatting of bibliographic references according to the journal’s requirements, and the creation and refinement of figures and graphical illustrations.

These tools were used exclusively as supportive instruments and not as autonomous sources of scientific evidence. All scientific statements, bibliographic references, and AI-assisted content were critically reviewed, verified against the original scientific literature where applicable, and edited by the authors. The authors retain full responsibility for the accuracy, integrity, scientific content, and conclusions of the manuscript.

## 3. Epidemiological and Clinical Evidence Linking Atopic Dermatitis to Bone Fragility

### 3.1. Evidence from Systematic Reviews and Meta-Analyses

The relationship between AD and skeletal health has been evaluated through several systematic reviews and meta-analyses, although their conclusions must be interpreted in the context of marked clinical and methodological heterogeneity [12,13,23,31]. The included studies varied in age distribution, AD definition, disease severity, duration of follow-up, corticosteroid exposure, and methods for identifying osteoporosis or fractures. Some investigations used DXA-derived measurements, whereas others relied on diagnostic codes, insurance claims, electronic health records, or self-reported outcomes. Consequently, the available evidence describes a consistent epidemiologic signal but does not establish a uniform biological effect across all patients with AD.

A 2021 systematic review concluded that AD may be associated with impaired bone health across different age groups but emphasized the limited number of studies with direct BMD measurements and adequate control of disease severity, medication exposure, and lifestyle-related confounders [12]. A contemporaneous meta-analysis found that adults with AD had higher odds of osteoporosis (OR 1.95, 95% CI 1.18–3.23), osteopenia (OR 1.90, 95% CI 1.51–2.38), and fracture (OR 1.13, 95% CI 1.05–1.22) than individuals without AD [13]. Nevertheless, the primary studies included in that analysis were heterogeneous and frequently differed in their definitions of AD and skeletal outcomes.

A subsequent meta-analysis with trial sequential analysis supported an association between AD and fracture but similarly noted that the available evidence was derived predominantly from observational datasets [23]. More recently, a 2026 meta-analysis restricted to cohort studies included 10 studies involving between several hundred and more than 2 million patients with AD [31]. The pooled analysis indicated an increased likelihood of osteoporosis (OR 1.56, 95% CI 1.14–2.13) and a smaller increase in the likelihood of any fracture (OR 1.08, 95% CI 1.05–1.10). However, heterogeneity was considerable for both osteoporosis and fracture outcomes, with I^2^ values of 99.9% and 82.1%, respectively [31]. These estimates therefore represent average associations across clinically diverse populations rather than a uniform risk applicable to every patient with AD.

The discrepancy between the relatively stronger association with osteoporosis and the more modest association with fracture is clinically relevant. Fracture is determined not only by BMD but also by bone microarchitecture, age, falls, muscle function, medication use, trauma exposure, and competing comorbidities. Conversely, osteoporosis diagnoses in administrative databases may be influenced by healthcare utilization and the likelihood of undergoing DXA. The two outcomes should therefore remain analytically distinct rather than being treated as interchangeable manifestations of a single AD-associated skeletal phenotype.

The substantial statistical heterogeneity observed in the available meta-analyses, particularly for osteoporosis (I^2^ = 99.9%) and overall fracture risk (I^2^ = 82.1%), limits the generalizability of pooled estimates [31]. Differences in age distribution, disease severity, cumulative glucocorticoid exposure, diagnostic definitions, and adjustment for potential confounders may contribute to this variability. Consequently, these findings should be interpreted as epidemiological associations rather than evidence of a direct causal effect of AD-related inflammation on skeletal deterioration. Although osteoimmunological mechanisms provide biological plausibility, their independent contribution to clinically relevant bone loss and fractures remains unconfirmed.

### 3.2. Bone Mineral Density and Osteoporosis Across the Lifespan

Evidence concerning BMD is not uniform across age groups. This is particularly important because AD often begins before peak bone mass is achieved but may persist into later adulthood, when age-related bone loss becomes increasingly relevant. The interpretation of low BMD must therefore account for growth, sex, menopausal status, body composition, and the appropriate use of Z-scores or T-scores.

In young adults, the available evidence does not support a generalized reduction in BMD among all individuals with AD. In a Korean population-based analysis of men younger than 50 years and premenopausal women, the overall prevalence of low BMD, defined by a Z-score of −2.0 or lower, did not differ significantly between participants with and without AD. Lumbar-spine BMD was modestly lower among men with AD, but low BMD was not more prevalent in the AD group as a whole. Within the AD population, early disease onset, longer disease duration, lower body mass index, lower vitamin D concentrations, and selected socioeconomic or reproductive factors were associated with lower BMD [16]. These results argue against considering AD alone sufficient to identify young adults at high skeletal risk and instead support a risk-enrichment model based on disease history and conventional bone-related factors.

Additional hypothesis-generating evidence was presented in meeting abstract P1067, which reported a 12-month cross-sectional comparison of 120 adults with moderate-to-severe AD and 120 healthy controls. Osteoporosis was identified in 25% of patients with AD versus 10% of controls, while osteopenia was reported in 40% versus 22%, respectively. Longer disease duration, higher Eczema Area and Severity Index scores, and systemic corticosteroid exposure were associated with less favorable skeletal outcomes. However, because these findings were reported only in abstract form, with limited information regarding participant selection, confounder adjustment, DXA interpretation, and model specification, they should be considered preliminary and require confirmation in a full peer-reviewed publication [70].

Pediatric studies present an additional interpretive challenge. Fractures in children are commonly traumatic and cannot automatically be classified as fragility fractures. Moreover, skeletal growth, pubertal development, nutritional status, physical activity, and body size strongly influence BMD. Observational studies have nevertheless identified a small increase in fracture incidence among children with AD [18,20]. In a Korean matched cohort, children with AD had an approximately 8% higher adjusted risk of fracture than unaffected children, with associations reported for skull and facial, truncal, and distal-limb fractures [18]. Systemic corticosteroid prescriptions, antihistamine exposure, and infant feeding characteristics were among the factors that contributed to the observed relationship. These findings suggest that childhood fracture risk may reflect a combination of disease severity, medication exposure, behavior, nutrition, and accidental injury rather than isolated deterioration of bone strength.

The contribution of topical corticosteroids remains uncertain but appears less concerning than that of systemic therapy. A pediatric population-based study found no convincing evidence that topical corticosteroid exposure independently increased fracture risk [19]. In adults, a randomized comparison of topical corticosteroid and tacrolimus treatment evaluated insulin sensitivity and bone homeostasis without establishing a clinically important adverse skeletal effect attributable to appropriately used topical corticosteroids [21]. Nevertheless, cumulative potency, treated body-surface area, exposure duration, adherence, and the potential for systemic absorption remain difficult to quantify in retrospective datasets.

Among older adults, the distinction between BMD and fracture becomes particularly evident. In a retrospective study of patients aged at least 45 years who underwent DXA, BMD was similar between patients with AD and matched controls. AD was not significantly associated with fractures at all skeletal sites (adjusted HR 2.55, 95% CI 0.72–9.01), but an association was observed for incident vertebral fractures (adjusted HR 6.80, 95% CI 1.77–26.17) during a relatively short follow-up period [24]. The wide confidence intervals, small AD sample size, and limited number of events limit the precision of this estimate. Nevertheless, the coexistence of similar BMD and greater vertebral fracture incidence raises the possibility that DXA alone may not capture all relevant aspects of skeletal vulnerability in older patients with AD.

### 3.3. Fracture Risk and Anatomical Patterns

Population-based studies generally support a modestly increased fracture risk in AD, but the magnitude varies according to disease severity, age, anatomical site, and study design [15,17,20,24,27,30,31]. In a nationwide matched cohort, the 16-year cumulative incidence of fracture was 8.043% in patients with AD and 7.366% in controls [15]. Severe AD was independently associated with fracture (adjusted HR 1.31, 95% CI 1.08–1.59), suggesting that fracture risk may be concentrated among patients with a greater cumulative inflammatory or treatment burden.

The most recent cohort-based meta-analysis found that the association varied by fracture site [31]. Vertebral fractures showed a pooled OR of 1.14 (95% CI 1.08–1.20), whereas lower-limb fractures had an OR of 1.11 (95% CI 1.08–1.13). These differences may reflect variations in trabecular and cortical bone vulnerability, falls, mobility, trauma patterns, and outcome ascertainment. However, the modest effect sizes and residual heterogeneity do not support using the fracture site alone to infer an AD-specific skeletal mechanism.

Large healthcare database studies have strengthened temporal evidence by identifying fracture events after AD diagnosis [17,20,27,30]. The longitudinal nature of these investigations reduces, but does not eliminate, concerns regarding reverse causation. Diagnostic coding cannot consistently distinguish low-energy fragility fractures from high-energy traumatic injuries, and administrative records frequently lack DXA values, bone turnover markers, cumulative inflammatory activity, falls, and detailed medication adherence. Increased healthcare contact among patients with AD may also raise the probability that osteoporosis or minor fractures are diagnosed and recorded.

Recent studies in adults and older populations further indicate that AD-related skeletal risk cannot be interpreted independently of the broader comorbidity profile [24,29,30]. Patients with persistent or severe AD may have a higher prevalence of sleep disturbance, depression, reduced physical activity, obesity, smoking, cardiovascular disease, and use of medications that influence falls or bone metabolism. A prospective UK Biobank investigation of allergic diseases also demonstrated that skeletal associations were not uniform across atopic conditions, with the strongest signals observed for asthma and cumulative allergic disease burden rather than for every allergic phenotype individually [32]. This reinforces the need to avoid extrapolating findings from one allergic disorder to another without disease-specific analyses.

### 3.4. Disease Severity, Duration, and Treatment Exposure

Disease severity appears to be one of the most reproducible modifiers of fracture risk. Cohort studies and meta-analytic subgroup analyses have reported progressively greater fracture risk in patients with more severe AD [15,17,22,30,31]. However, disease severity is commonly defined indirectly through prescriptions, specialist consultations, hospital diagnoses, or use of systemic treatment. These proxies may capture both inflammatory burden and treatment intensity, making their individual contributions difficult to separate.

Duration and age at onset may also be important. In young adults, early-onset and longer-lasting AD were associated with low BMD within the affected population, even though AD was not associated with a higher overall prevalence of low BMD relative to controls [16]. Long-standing disease may influence skeletal health through cumulative inflammation, repeated glucocorticoid exposure, prolonged sleep disturbance, physical inactivity, nutritional restriction, or failure to achieve optimal peak bone mass. Nevertheless, longitudinal studies beginning in childhood and incorporating repeated DXA examinations are lacking.

Systemic glucocorticoid exposure is an established cause of secondary osteoporosis and represents a major potential mediator of the AD–fracture relationship [25,26,48,49,50]. In the nationwide cohort reported by Lin et al., both topical corticosteroid exposure and systemic corticosteroid doses of at least 10 mg/day were associated with fracture, with a stronger association for systemic treatment [15]. Such results must be interpreted cautiously because treatment intensity is also a marker of disease severity.

Importantly, a population-based analysis specifically evaluating oral corticosteroids concluded that only a limited proportion of the association between atopic eczema and major osteoporotic fracture was explained by oral corticosteroid exposure [17]. This finding suggests that systemic glucocorticoids are clinically important but unlikely to represent the sole explanation for the observed epidemiologic association. In contrast, appropriately used topical corticosteroids have not consistently been associated with fracture in pediatric cohorts [19]. The overall evidence therefore supports distinguishing systemic exposure from topical treatment rather than grouping all corticosteroid use into a single risk category.

Recent claims-based analyses confirm that systemic glucocorticoids are still used in AD, despite guideline recommendations favoring more targeted long-term strategies [25,26]. Their harms are likely to depend on cumulative dose, treatment duration, frequency of repeated courses, baseline fracture risk, age, menopausal status, and concomitant medications. Studies that classify exposure only as present or absent may therefore underestimate clinically relevant dose–response relationships.

The principal clinical studies evaluating bone mineral density, osteoporosis, and fracture outcomes in patients with AD are summarized in Table 1.

Collectively, these studies support a modest but clinically relevant association between AD and bone fragility, while substantial methodological heterogeneity and the influence of disease severity, age, treatment exposure, and conventional skeletal risk factors preclude a simple causal interpretation.

### 3.5. Clinical Interpretation of the Epidemiologic Evidence

Taken together, current evidence supports an association between AD and skeletal outcomes, but the effect is heterogeneous and generally modest at the population level. The most defensible interpretation is not that AD uniformly causes osteoporosis, but that it may contribute to a multifactorial skeletal-risk phenotype in susceptible subgroups. Patients with severe or long-standing disease, repeated systemic glucocorticoid exposure, low body mass index, nutritional restriction, low vitamin D status, reduced physical activity, sleep disturbance, older age, or additional conventional osteoporosis risk factors are likely to account for a substantial proportion of the observed burden.

Several findings argue against an overly simplified disease-to-bone pathway. Low BMD was not more prevalent among all young adults with AD [16]; older patients may experience vertebral fractures despite BMD values similar to those of controls [24]; oral corticosteroids explained only part of the fracture association [17]; and the magnitude of risk varied substantially across fracture sites, age groups, and severity strata [18,20,31]. These observations suggest that BMD, bone quality, falls, trauma, inflammation, treatment, and lifestyle may contribute differently throughout the lifespan.

The epidemiologic literature also does not currently justify universal DXA screening solely on the basis of an AD diagnosis. Instead, the evidence supports targeted skeletal assessment in patients whose AD coexists with established clinical risk factors or indicators of cumulative disease burden. This risk-adapted interpretation is consistent with the modest pooled fracture estimate, the pronounced between-study heterogeneity, and the absence of validated AD-specific screening thresholds.

Future studies should combine standardized AD severity measurements with cumulative treatment exposure, repeated DXA, age-appropriate Z-score or T-score interpretation, trabecular bone score or other measures of bone quality, standardized bone turnover markers, falls assessment, and adjudicated low-trauma fractures. Prospective designs should also evaluate whether effective long-term control of AD reduces skeletal risk through improved mobility, sleep, nutrition, and reduced systemic glucocorticoid exposure. Until such evidence becomes available, the association between AD and bone fragility should be regarded as clinically relevant but heterogeneous, multifactorial, and not yet proven to be directly causal.

## 4. Osteoimmunological Mechanisms Linking Atopic Dermatitis to Bone Remodeling

### 4.1. Bone Remodeling as an Immune-Regulated Process

Bone is a metabolically active tissue maintained through continuous coupling between osteoclastic resorption and osteoblastic formation. Osteocytes coordinate much of this process by sensing mechanical and hormonal signals and by regulating the availability of RANKL. Binding of RANKL to its receptor RANK on osteoclast precursors activates signaling pathways—including NF-κB, mitogen-activated protein kinases, and nuclear factor of activated T cells 1—that promote osteoclast differentiation, survival, and bone-resorptive activity. OPG, a soluble decoy receptor produced primarily by cells of the osteoblastic lineage, prevents RANKL–RANK interaction and thereby restrains osteoclastogenesis. Consequently, the balance between RANKL and OPG is more informative than either mediator considered in isolation [33,35,37,43].

The immune and skeletal systems share numerous cellular populations and signaling mediators. Activated T cells, B cells, macrophages, dendritic cells, and stromal cells can influence RANKL expression or produce cytokines that regulate osteoclast and osteoblast activity. Pro-inflammatory mediators such as tumor necrosis factor-α (TNF-α), IL-1β, IL-6, and IL-17 generally favor osteoclastogenesis, whereas other cytokines may exert anti-resorptive, anabolic, or context-dependent effects. Chronic inflammatory diseases can therefore impair bone strength by shifting remodeling toward resorption, suppressing bone formation, or disrupting the normal coupling of these processes [34,37,43].

AD differs immunologically from classic osteoclastogenic disorders such as rheumatoid arthritis. Its dominant cytokine profile is usually type 2-skewed, particularly during the acute phase, although chronic and severe disease may involve broader immune activation, including type 1, type 17, and type 22 pathways. The skeletal consequences of AD cannot therefore be inferred from a single cytokine or from a simple “inflammation causes bone loss” model. Instead, they probably reflect the combined effects of disease phenotype, inflammatory duration, age, hormonal environment, treatment exposure, nutrition, physical activity, and mechanical loading.

### 4.2. The Context-Dependent Effects of IL-4 and IL-13

IL-4 and IL-13 are central mediators of type 2 inflammation in AD. Both signal through receptor complexes containing the IL-4 receptor-α subunit, activate Janus kinase–signal transducer and activator of transcription pathways, and contribute to epidermal barrier dysfunction, pruritus, microbial susceptibility, and chronic cutaneous inflammation. Their effects on bone, however, are biologically complex.

Experimental studies have frequently shown that IL-4 can suppress osteoclast differentiation by interfering with RANKL-induced signaling and by promoting an alternatively activated, anti-inflammatory macrophage phenotype. IL-4 has also reduced osteolysis in models of cartilage-debris inflammation, wear-particle disease, and periodontitis [38,39,44]. IL-13 may similarly inhibit bone erosion under certain inflammatory conditions, partly through increased OPG expression and attenuation of osteoclastogenic signaling [42]. These observations challenge the assumption that type 2 cytokines are intrinsically detrimental to bone.

Their effects nevertheless depend on cytokine concentration, exposure duration, tissue compartment, cellular differentiation stage, and the surrounding inflammatory milieu. In a model of food-allergic enteropathy, an excessive IL-4 environment enhanced osteoclastogenesis and altered inflammatory-cell differentiation, illustrating that sustained type 2 polarization may become osteoclastogenic under specific biological conditions [47]. Moreover, cytokines that inhibit isolated osteoclast precursors in vitro may produce different net effects in vivo when acting simultaneously on osteoblasts, stromal cells, immune populations, intestinal absorption, endocrine pathways, and skeletal muscle.

Accordingly, the association between AD and osteoporosis should not be attributed directly to elevated IL-4 or IL-13 concentrations. Current evidence supports a bidirectional and context-dependent model in which type 2 signaling may be locally anti-osteoclastogenic in some settings but contribute indirectly—or occasionally directly—to skeletal deterioration when excessive, persistent, or accompanied by other inflammatory and metabolic disturbances [34,37,43,46,47].

### 4.3. The IL-33/IL-31 Axis: A Potential Interface Between Pruritus, Inflammation, and Bone

IL-33 is an epithelial alarmin released in response to tissue stress or damage and is implicated in the initiation and amplification of type 2 immune responses. In AD, IL-33 can activate group 2 innate lymphoid cells, mast cells, eosinophils, and T-helper 2 cells. It may also promote IL-31 production, thereby linking epithelial damage and immune activation with pruritus. Because IL-31 is strongly associated with itch and sleep disturbance, the IL-33/IL-31 axis provides a plausible interface between cutaneous inflammation, neuroimmune signaling, behavioral changes, and skeletal health [36].

The direct skeletal actions of this axis remain incompletely defined. IL-33 has inhibited RANKL-dependent osteoclastogenesis in several experimental systems, and its administration or preserved signaling has reduced bone resorption in selected models of inflammatory or mechanically induced bone loss [36,40,41]. Conversely, circulating IL-31 has been associated with reduced bone mineral density in osteoporosis, and prolonged IL-31-dominant signaling has been proposed to promote osteoclastogenesis. The biological relationship may therefore involve a regulatory imbalance: IL-33 may exert anti-resorptive effects in bone while simultaneously promoting type 2 inflammation and IL-31 production in immune tissues [36].

Translation of these observations to AD requires caution. Much of the available evidence derives from osteoporosis cohorts, periodontal models, or experimental systems rather than from patients with AD undergoing longitudinal skeletal assessment. Moreover, circulating cytokine concentrations may not accurately represent signaling within skin, bone marrow, or the bone-remodeling compartment. The IL-33/IL-31 axis should consequently be regarded as a mechanistically credible research pathway rather than a clinically validated mediator of AD-associated fractures.

### 4.4. Inflammation Beyond the Type 2 Axis

Although IL-4 and IL-13 dominate many AD phenotypes, chronic disease is immunologically heterogeneous. Persistent barrier disruption, microbial dysbiosis, repeated infection, and tissue injury can recruit additional inflammatory pathways. Cytokines such as TNF-α, IL-1β, IL-6, and IL-17 may increase RANKL expression, amplify osteoclast differentiation, and suppress osteoblast function, particularly when inflammation becomes sustained or systemic [37,43].

Experimental evidence further suggests that systemic type 2 inflammation associated with AD can aggravate inflammatory bone destruction outside the skin. In an AD-associated model of periodontitis, systemic type 2 inflammation intensified periodontal disease and local tissue damage [45]. This observation supports the existence of biologically relevant communication between inflamed skin and distant musculoskeletal or periodontal tissues. However, periodontal bone loss is driven by a highly specialized microbial and mechanical environment and cannot be considered equivalent to generalized osteoporosis. The findings demonstrate systemic inflammatory potential but do not establish that cutaneous inflammation directly causes axial or appendicular bone loss in humans.

AD may also affect skeletal health through inflammatory effects on muscle. Chronic inflammation, sleep fragmentation, pain, and reduced physical activity may diminish muscle mass or performance, reduce mechanical stimulation of bone, and increase fall susceptibility. RANK–RANKL–OPG signaling participates in muscle–bone communication as well as osteoclast regulation, further emphasizing that fracture risk cannot be reduced to bone mineral density alone [33]. Nevertheless, direct evidence linking AD-specific cytokine profiles to sarcopenia, impaired bone–muscle crosstalk, and incident fragility fractures remains limited.

### 4.5. Growth, Peak Bone Mass, and Lifespan Considerations

The clinical significance of inflammatory interference with bone remodeling varies across the lifespan. During childhood and adolescence, impaired linear growth, delayed skeletal maturation, dietary restriction, low body mass, reduced physical activity, and systemic glucocorticoid exposure may prevent attainment of optimal peak bone mass. Even modest disturbances sustained during critical growth periods may therefore influence skeletal reserve later in life. The potential roles of IL-4 and IL-13 signaling in growth and osteoimmunology have attracted particular interest, but current evidence does not establish that pharmacological inhibition of these cytokines improves final height or peak bone mass [46].

In adults, persistent inflammation and cumulative treatment exposure may accelerate remodeling imbalance or interact with reproductive, endocrine, and lifestyle determinants. In older adults, the same disease-related influences are superimposed on age-related bone loss, sarcopenia, impaired balance, polypharmacy, and increased fall risk. Thus, a comparable inflammatory exposure may have different skeletal consequences in a child acquiring bone mass, a young adult maintaining peak bone mass, and an older adult experiencing accelerated cortical and trabecular deterioration.

These age-dependent mechanisms also affect interpretation of dual-energy X-ray absorptiometry. In growing individuals, reduced bone size or delayed maturation can lower areal bone mineral density without necessarily indicating the same biological process as postmenopausal osteoporosis. Mechanistic hypotheses must therefore be integrated with age-appropriate densitometric interpretation rather than inferred solely from an isolated low measurement.

### 4.6. An Integrated Mechanistic Model

An important distinction should be made between experimental evidence supporting the biological plausibility of immune–bone interactions and direct clinical evidence in patients with AD. The effects of IL-4, IL-13, IL-31, and IL-33 on osteoclast differentiation, osteoblast activity, and RANK–RANKL–OPG signaling have been investigated predominantly in cell cultures, animal models, or inflammatory conditions other than AD [34,36,38,39,40,41,42,44,45,46,47]. In contrast, direct clinical evidence in AD remains limited. The association between disease severity and circulating RANKL/OPG levels reported by Sakai et al. [14] provides a relevant clinical observation, but its cross-sectional design precludes causal inference. Furthermore, circulating biomarkers do not necessarily reflect bone-tissue remodeling or predict future fractures. Thus, the proposed osteoimmunological mechanisms should be regarded as biologically plausible hypotheses rather than established causal pathways linking AD to osteoporosis.

Available evidence supports a multilevel model rather than a single causal pathway. First, chronic cutaneous and systemic immune activation may alter RANKL–RANK–OPG signaling and the balance between osteoclast and osteoblast activity. Second, the skeletal effects of IL-4, IL-13, IL-31, and IL-33 are context-dependent and may vary with cytokine concentration, disease chronicity, cellular environment, and coexisting inflammatory signals. Third, AD can indirectly influence bone through sleep disruption, reduced activity, nutritional restriction, low body mass, glucocorticoid exposure, muscle impairment, and falls. Finally, age and baseline skeletal reserve determine whether these exposures impair peak bone acquisition, accelerate adult bone loss, or translate into fractures.

This framework reconciles the apparently paradoxical evidence that type 2 cytokines may inhibit osteoclastogenesis experimentally while patients with severe AD can still demonstrate increased skeletal risk. The paradox does not require IL-4 or IL-13 to act as universal osteoclastogenic mediators. Instead, the net clinical phenotype emerges from interactions among immune signaling, treatment, behavior, nutrition, endocrine status, muscle function, and the mechanical environment.

**Figure 1:**
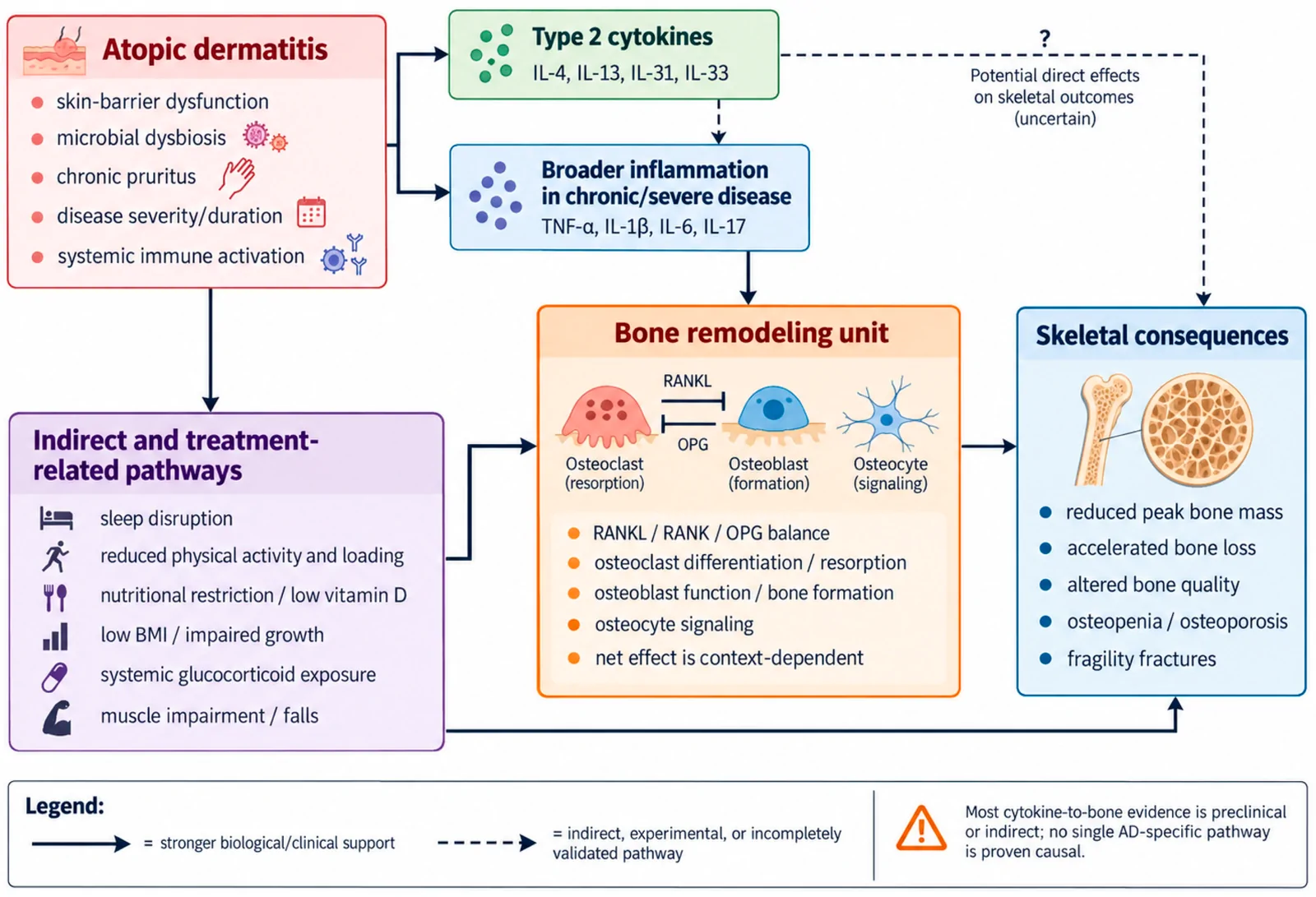
**Mechanistic osteoimmunological framework linking atopic dermatitis to bone fragility.** Atopic dermatitis (AD) may contribute to bone fragility through multiple interacting pathways. Core disease features, including skin-barrier dysfunction, microbial dysbiosis, chronic pruritus, prolonged disease duration, and systemic immune activation, may promote type 2 cytokine signaling (IL-4, IL-13, IL-31, IL-33), broader inflammatory activation in more severe disease (including TNF-α, IL-1β, IL-6, and IL-17), and indirect clinical consequences such as sleep disruption, reduced physical activity and skeletal loading, nutritional compromise, impaired growth or low BMI, systemic glucocorticoid exposure, and increased fall risk. These factors may converge on the bone remodeling unit by influencing the RANKL/RANK/osteoprotegerin (OPG) axis, osteoclast differentiation and resorption, osteoblast-mediated bone formation, and osteocyte signaling. The resulting skeletal consequences may include reduced peak bone mass, accelerated bone loss, altered bone quality, osteopenia or osteoporosis, and fragility fractures. Solid arrows indicate pathways with relatively stronger biological or clinical support, whereas dashed arrows indicate indirect, experimental, or incompletely validated mechanisms. Most cytokine-to-bone evidence remains preclinical or indirect, and no single AD-specific pathway has been definitively established as causal.

The proposed direct and indirect pathways linking AD to altered bone remodeling and increased skeletal fragility are depicted in Figure 1.

Importantly, no currently available human study has demonstrated a complete causal chain from AD-specific cytokine activity to altered bone remodeling, longitudinal bone loss, and subsequent fragility fracture. Most mechanistic evidence is preclinical, indirect, or extrapolated from other inflammatory conditions. Osteoimmunology therefore provides biological plausibility for the epidemiological association, but it does not by itself establish causality or justify cytokine-targeted therapy as a treatment for osteoporosis. Prospective studies that combine standardized AD activity measures, circulating and tissue biomarkers, bone turnover markers, age-appropriate imaging, treatment exposure, muscle assessment, and incident low-trauma fractures are needed to validate the proposed pathways.

## 5. Modifiable and Treatment-Related Determinants of Bone Fragility

The skeletal phenotype associated with atopic dermatitis is unlikely to result exclusively from cutaneous inflammation. AD commonly coexists with behavioral, nutritional, metabolic, and treatment-related exposures that may impair bone acquisition, accelerate bone loss, or increase the likelihood that reduced bone strength results in fracture. Some of these factors may act as confounders of the epidemiological association, whereas others may mediate or modify the effect of AD on skeletal outcomes. Their relative contribution varies according to disease severity, age, cumulative exposure, and baseline skeletal reserve.

### 5.1. Systemic Glucocorticoid Exposure

Systemic glucocorticoids represent the most clearly established treatment-related determinant of skeletal fragility. Although contemporary AD guidelines discourage their routine or prolonged use, oral or parenteral glucocorticoids continue to be prescribed for severe flares, rapid symptom control, or situations in which access to targeted therapies is limited. Repeated short courses may also produce substantial cumulative exposure even when no single treatment period is prolonged [25,26].

Glucocorticoids affect both components of bone remodeling. They suppress osteoblast differentiation, promote osteoblast and osteocyte apoptosis, reduce bone matrix synthesis, and impair maintenance of the osteocyte network. Simultaneously, they can prolong osteoclast survival during the initial phase of exposure, resulting in rapid bone loss. Additional effects include reduced intestinal calcium absorption, increased renal calcium loss, suppression of the gonadal and growth hormone axes, muscle wasting, and increased fall risk [49]. Fracture risk may consequently increase rapidly and exceed that predicted by BMD alone.

Current recommendations for glucocorticoid-induced osteoporosis emphasize early fracture-risk assessment in adults initiating or continuing prednisone-equivalent doses of at least 2.5 mg/day for more than three months. Assessment should consider dose, duration, and pattern of exposure; previous fractures; age; body weight; smoking and alcohol use; hypogonadism; falls; and other causes of secondary osteoporosis. In adults aged ≥40 years, clinical evaluation can be combined with FRAX and DXA, including vertebral fracture assessment or spinal imaging when indicated. In younger adults, children, and adolescents, interpretation must account for age, growth, pubertal development, and the limitations of conventional fracture-risk algorithms [48,50].

These principles are directly relevant to patients with AD who receive repeated or prolonged systemic glucocorticoids. However, systemic therapy does not appear to explain the entire AD–fracture association. Population-based analyses have reported residual fracture risk after adjustment for oral corticosteroid exposure, while topical corticosteroid studies have generally been more reassuring [17,19,21]. Systemic glucocorticoids should therefore be considered an important and preventable component of risk rather than the sole causal explanation.

The interaction between disease activity and treatment exposure is illustrated by meeting abstract P1066, which described a 38-year-old woman with a 25-year history of AD, increasing corticosteroid use, reduced physical activity, back and hip pain, and DXA-defined osteoporosis. Elevated bone-turnover markers were reported despite normal calcium and vitamin D concentrations. Although an individual case cannot establish either the independent contribution of AD inflammation or a causal effect of corticosteroids, it illustrates the cumulative clinical phenotype in which long disease duration, repeated treatment exposure, and inactivity coexist [71].

Topical corticosteroids should not be grouped automatically with systemic glucocorticoids. Systemic absorption varies with potency, treated surface area, treatment duration, occlusion, age, and barrier integrity, but routine topical use generally results in substantially lower exposure. Current evidence does not support avoiding appropriate topical anti-inflammatory treatment because of an assumed fracture risk. Inadequately controlled AD could itself increase systemic inflammation, sleep disturbance, physical inactivity, and the subsequent requirement for systemic rescue therapy.

### 5.2. Vitamin D Status: Shared Marker, Potential Mediator, or Therapeutic Target?

Vitamin D is relevant to both epidermal and skeletal biology. It contributes to calcium and phosphate homeostasis, bone mineralization, immune regulation, keratinocyte differentiation, antimicrobial defense, and maintenance of the epidermal barrier. Several characteristics of patients with AD—including reduced outdoor activity, sun avoidance, darker skin pigmentation, restrictive diets, obesity or low body mass, and chronic inflammation—may influence serum 25-hydroxyvitamin D concentrations.

Meta-analytic evidence indicates that lower serum 25-hydroxyvitamin D levels are associated with greater AD severity and that supplementation may modestly improve clinical severity scores in some populations [51,52,53]. However, these findings do not establish vitamin D deficiency as a cause of AD, nor do improvements in dermatitis severity demonstrate prevention of osteoporosis or fractures. Reverse causation is possible because patients with severe disease may spend less time outdoors, modify their diets, or differ systematically from those with mild disease.

The effects of vitamin D supplementation on AD have also been heterogeneous. Differences in baseline vitamin D status, age, latitude, season, dose, treatment duration, and clinical severity contribute to inconsistent results across trials [51,52,53]. Supplementation may be most rational when biochemical deficiency, insufficient intake, impaired absorption, or elevated skeletal risk is documented. Routine high-dose supplementation solely because a patient has AD is not supported by current evidence.

From a bone-health perspective, vitamin D should be assessed within the broader context of calcium intake, nutritional adequacy, renal and hepatic function, malabsorption, medication exposure, and fracture risk. Correction of deficiency is important for normal mineral metabolism, particularly in patients receiving systemic glucocorticoids, but vitamin D alone is insufficient treatment for individuals at high or very high fracture risk [48,50].

### 5.3. Dietary Restriction, Food Allergy, and Nutritional Adequacy

Food allergy and perceived food-triggered eczema frequently lead patients or caregivers to eliminate milk, eggs, wheat, nuts, or other nutrient-dense foods. When medically indicated and supervised, elimination diets may be necessary. Unstructured or prolonged restriction without confirmed food allergy, however, can reduce the intake of calcium, vitamin D, protein, phosphorus, magnesium, and total energy.

The skeletal impact may be particularly important during childhood and adolescence, when nutritional insufficiency can impair linear growth and peak bone-mass acquisition. Low weight or reduced lean mass may further decrease mechanical loading of bone. In adults and older individuals, inadequate protein and energy intake may contribute to sarcopenia, frailty, and falls. These consequences may occur even when DXA measurements do not yet meet conventional thresholds for osteoporosis.

Randomized dietary-intervention studies in children with AD but without food allergy have not established broad elimination diets as an effective routine treatment for cutaneous symptoms [54]. Dietary exclusions should therefore be based on an appropriate allergy evaluation rather than on AD alone. When major food groups are removed, nutritional monitoring and replacement of calcium, vitamin D, protein, and other relevant nutrients should be considered.

Obesity may also complicate interpretation. Higher body weight can increase areal BMD through increased mechanical loading but may coexist with low vitamin D levels, poor diet quality, physical inactivity, and impaired balance. Conversely, low BMI is a recognized fracture-risk factor and was associated with less favorable BMD among young adults with AD [16]. Body weight should thus be interpreted alongside body composition, muscle function, nutritional status, and age, rather than as an isolated protective or harmful characteristic.

### 5.4. Physical Activity, Mechanical Loading, and Muscle–Bone Interactions

Weight-bearing activity and muscle contraction are essential stimuli for bone acquisition and maintenance. Physical activity also improves balance, coordination, and muscle strength, thereby reducing the risk of fall-related fractures. AD may interfere with these protective mechanisms through pruritus, sweating-induced discomfort, visible lesions, embarrassment, sleep deprivation, or avoidance of sports and outdoor activities.

Adolescents with AD have demonstrated lower peak exercise-load capacity and lower exercise volume than unaffected peers [55]. Although these findings do not demonstrate reduced BMD or increased fracture risk, they identify a plausible pathway through which persistent disease during growth could diminish bone-loading stimuli and compromise cardiometabolic and musculoskeletal fitness. Reduced exercise may be especially relevant in patients with severe disease, obesity, asthma, sleep disturbance, or psychological comorbidity.

Lifestyle studies also suggest that some patients with AD have suboptimal patterns of physical activity and other health behaviors [56]. Nevertheless, the available evidence is primarily cross-sectional and susceptible to reverse causation: severe AD may reduce activity, while physical inactivity may worsen general health without directly influencing cutaneous disease. Objective longitudinal studies that combine accelerometry, muscle strength testing, body composition, DXA, and fracture outcomes are needed.

Clinical advice should emphasize individualized, tolerable weight-bearing and resistance activities. Disease control, breathable clothing, appropriate use of emollients, temperature regulation, and prompt removal of sweat may help patients remain active. Exercise recommendations should account for age, baseline fitness, fracture history, systemic glucocorticoid exposure, and coexisting musculoskeletal disease.

### 5.5. Pruritus, Sleep Disturbance, and Falls

Sleep disturbance is one of the most burdensome systemic consequences of AD. Nocturnal pruritus, scratching, altered skin temperature, and circadian variation in inflammatory mediators can delay sleep onset and cause repeated awakenings. Sleep impairment may persist even when visible disease activity improves and can affect both patients and caregivers [57].

Several indirect mechanisms could link sleep disturbance to skeletal fragility. Chronic sleep restriction may alter endocrine and metabolic pathways relevant to bone turnover, reduce daytime physical activity, impair muscle recovery, and increase fatigue. In older adults, nocturnal awakenings, sedating antihistamines, anxiolytics, antidepressants, or other centrally acting medications may increase instability and fall risk. Scratching-related awakenings, followed by walking in darkness, may pose an additional practical hazard.

However, direct evidence demonstrating that AD-related sleep disturbance causes osteoporosis or fractures is currently lacking. Sleep should therefore be considered a clinically relevant potential mediator and fall-risk modifier rather than a proven mechanism of bone loss. Assessment may include pruritus severity, nocturnal awakenings, daytime somnolence, sedating medication use, balance impairment, and previous falls, particularly in older or frail patients.

### 5.6. Smoking, Alcohol, and Other Lifestyle Factors

Smoking and excessive alcohol consumption are established risk factors for osteoporosis and fractures in the general population. They may influence bone through impaired osteoblast function, altered calcium and vitamin D metabolism, endocrine effects, reduced muscle quality, and increased susceptibility to falls. These exposures should be incorporated into skeletal-risk assessment irrespective of whether they are more prevalent among patients with AD.

The relationship between AD and lifestyle behavior is heterogeneous. Psychological distress, anxiety, depression, sleep disturbance, and reduced quality of life may promote unhealthy coping behaviors in some patients, whereas health-conscious behavior may reduce exposure in others [56]. Observational associations must therefore be interpreted without assuming that AD directly causes smoking, alcohol consumption, or physical inactivity.

Other relevant factors include low calcium and protein intake, limited sunlight exposure, prolonged immobilization, early menopause, hypogonadism, thyroid or parathyroid disease, malabsorption, chronic kidney or liver disease, and concurrent medications that impair bone or increase falls. These conventional determinants may contribute more strongly to an individual patient’s absolute fracture risk than AD itself.

### 5.7. Shift Work, Occupational Stress, and Circadian Disruption

Shift work may represent an underrecognized modifier of skeletal risk in patients with AD. Rotating three-shift schedules, night work, irregular recovery periods, and occupational stress can disrupt circadian organization, reduce sleep duration and quality, alter meal timing, and limit opportunities for regular physical activity and sunlight exposure. These effects may compound several of the behavioral and metabolic pathways already implicated in both AD severity and impaired bone health.

For patients with AD, working at night or on rapidly rotating schedules may be particularly relevant because pruritus and sleep fragmentation can already interfere with restorative sleep [57]. Circadian misalignment, occupational stress, heat exposure, sweating, protective clothing, and limited access to appropriate skin care during working hours may additionally contribute to poorer disease control. In turn, worsening symptoms may increase fatigue, reduce exercise tolerance, and promote intermittent use of systemic glucocorticoids or sedating medications.

Irregular work schedules may also affect nutritional behavior. Skipped meals, eating during the biological night, reduced dietary quality, and limited outdoor activity could contribute to inadequate calcium or vitamin D intake, unfavorable body composition, and reduced mechanical loading of bone. The combination of nocturnal work, sleep deprivation, and sedating medication may be especially important in occupations involving physical hazards, because it could increase fall and injury risk independently of BMD.

Nevertheless, direct evidence specifically linking shift work to osteoporosis or fragility fractures in patients with AD is currently lacking. Shift work should therefore be regarded as a potential contextual and occupational risk modifier rather than an established AD-specific determinant of bone loss. In clinical practice, occupational history may be considered in patients with moderate-to-severe AD, particularly when rotating or night work coexists with persistent sleep disturbance, low physical activity, nutritional irregularity, systemic glucocorticoid exposure, or previous falls [56,57].

### 5.8. A Cumulative Risk Model

The modifiable determinants of skeletal fragility in AD are likely to operate cumulatively. A young patient with mild, well-controlled disease, adequate nutrition, normal growth, regular physical activity, and no systemic glucocorticoid exposure is unlikely to have the same skeletal risk as an older patient with severe long-standing AD, repeated oral glucocorticoid courses, low BMI, vitamin D deficiency, impaired sleep, sarcopenia, and previous falls.

These exposures can also reinforce one another. Severe pruritus may disrupt sleep, which reduces daytime activity and muscle performance. Lower activity decreases mechanical loading of bone, while systemic glucocorticoids can further impair bone formation and muscle strength. Restrictive diets may compound low body mass and vitamin D deficiency. In older adults, the combined effect may increase both skeletal vulnerability and the likelihood of falling.

A similar cumulative pattern was described in [72]. A 40-year-old woman with 15 years of moderate-to-severe AD, recurrent oral corticosteroid treatment, reduced physical activity, and ongoing systemic inflammation was found to have lumbar-spine osteoporosis. The authors attributed the skeletal phenotype to the combined effects of chronic disease activity, corticosteroid exposure, and reduced mechanical loading. This interpretation is clinically plausible but remains descriptive because the relative contribution of each factor could not be determined from a single case [72].

This cumulative model has two clinical implications. First, the presence of AD alone should not automatically trigger a diagnosis of osteoporosis or universal DXA screening. Second, bone-health assessment should not be limited to systemic glucocorticoid use. A risk-adapted approach should integrate disease severity and duration with age, fracture history, cumulative treatment exposure, nutritional status, body composition, physical activity, sleep, falls, and conventional osteoporosis risk factors. Such an approach is more consistent with the multifactorial evidence than either attributing skeletal risk entirely to inflammation or dismissing it as a consequence of glucocorticoid therapy alone.

## 6. Targeted Systemic Therapy and the Bone Paradox

The expansion of biologic and small-molecule therapies has transformed the management of moderate-to-severe atopic dermatitis. This therapeutic shift is also relevant to bone health because effective long-term disease control may reduce systemic inflammation, pruritus, sleep disruption, physical inactivity, nutritional impairment, and exposure to systemic glucocorticoids. Conversely, targeted inhibition of cytokines involved in bone remodeling raises theoretical concerns about unintended skeletal effects. The resulting balance between improved disease control and direct immunological modulation constitutes a therapeutic paradox that is particularly relevant to dupilumab.

### 6.1. From Broad Immunosuppression to Targeted Disease Control

Conventional systemic treatments for AD include systemic glucocorticoids, cyclosporine, methotrexate, azathioprine, and mycophenolate mofetil. These agents differ substantially in mechanism, treatment duration, monitoring requirements, and toxicity. Among them, systemic glucocorticoids have the clearest adverse skeletal profile, whereas evidence regarding the long-term effects of other conventional immunosuppressants on BMD or fracture risk in AD remains limited.

Current American and European guidelines recommend targeted systemic therapies for eligible patients with moderate-to-severe disease and advise against the routine use of systemic glucocorticoids [60,61]. From a skeletal perspective, this transition may be beneficial even if a targeted drug has no direct anabolic effect on bone. A glucocorticoid-sparing strategy can reduce one of the most clinically important modifiable contributors to secondary osteoporosis while improving disease control sufficiently to restore sleep, physical activity, nutrition, and quality of life.

However, evidence that a therapy improves AD should not automatically be interpreted as evidence that it prevents osteoporosis [58,60,61,62,64]. Most pivotal trials and long-term extension studies were designed primarily around cutaneous endpoints, including the Eczema Area and Severity Index, Investigator’s Global Assessment, pruritus scores, and quality-of-life measures [58,60,61,62,64]. With the notable exception of a recent post hoc pediatric analysis evaluating linear growth and bone-related biomarkers [59], DXA, vertebral imaging, bone microarchitecture, bone turnover markers, falls, and fragility fractures were generally not prespecified as outcomes. Consequently, the comparative skeletal effects of systemic AD therapies remain largely unknown [60,61,64].

### 6.2. Dupilumab and the IL-4/IL-13 Signaling Paradox

Dupilumab is a fully human monoclonal antibody directed against the IL-4 receptor-α subunit, thereby inhibiting signaling by both IL-4 and IL-13. It is strongly recommended for adults with moderate-to-severe AD who are candidates for systemic therapy and is approved across a broad pediatric age range [60,61]. Long-term extension and real-world studies have demonstrated sustained disease control with a generally consistent safety profile [58,62,64].

The theoretical bone paradox arises because IL-4 and IL-13 can inhibit osteoclastogenesis under several experimental conditions [38,39,42,44]. Blocking their signaling could therefore be hypothesized to remove an anti-resorptive influence. Such an interpretation, however, is overly simplistic. As discussed in Section 4, the skeletal actions of type 2 cytokines vary with concentration, timing, target-cell population, inflammatory environment, and experimental model [37,43,46,47]. Excessive or chronic type 2 inflammation may exert distinct net effects compared with physiological cytokine signaling, and findings from isolated bone-cell systems cannot be translated directly to patients with AD [34,37,43,46,47].

This theoretical concern was reflected in [73], which described a 52-year-old woman with long-standing AD who developed back pain and DXA-defined osteoporosis six months after initiating dupilumab. Normal calcium and vitamin D concentrations, along with elevated bone turnover markers, were reported. The temporal sequence prompted the authors to hypothesize that modulation of IL-4/IL-13 might influence bone remodeling. However, the absence of serial pretreatment DXA measurements, detailed baseline fracture-risk assessment, menopausal and hormonal characterization, and an untreated comparator prevents attribution of the skeletal finding to dupilumab [73].

In clinical practice, dupilumab simultaneously suppresses chronic inflammation, improves the epidermal barrier, reduces pruritus, restores sleep, and may enable greater physical activity and more adequate nutrition. It can also reduce the need for systemic glucocorticoids. These indirect effects could outweigh any theoretical consequence of IL-4/IL-13 inhibition within the bone-remodeling compartment. The net skeletal effect must therefore be established empirically rather than inferred solely from cytokine biology.

### 6.3. Adult Evidence: Long-Term Safety Without Dedicated Skeletal Endpoints

A five-year open-label extension study in adults with moderate-to-severe AD demonstrated sustained clinical effectiveness and a long-term safety profile consistent with previous dupilumab studies [58]. Importantly, osteoporosis and fractures did not emerge as established treatment-related safety concerns. Nevertheless, absence of a prominent safety signal is not equivalent to proof of skeletal neutrality or benefit.

The adult dupilumab program has not provided a comprehensive longitudinal assessment that incorporates baseline and follow-up DXA, vertebral fracture assessment, trabecular bone score, bone turnover biomarkers, falls, and adjudicated fragility fractures. Trial participants may also differ from patients encountered in routine practice, particularly older adults with pre-existing osteoporosis, prolonged systemic glucocorticoid exposure, multimorbidity, or frailty.

Consequently, current evidence from adults is reassuring but incomplete. There is no robust clinical evidence that dupilumab causes osteoporosis, accelerates bone loss, or increases the risk of fragility fractures. Equally, there is insufficient evidence to prescribe dupilumab specifically to improve BMD or prevent fractures.

### 6.4. Pediatric Evidence: Growth and Bone Biomarkers

The pediatric setting is particularly important because severe AD can occur during periods of rapid linear growth and peak bone-mass acquisition. Chronic inflammation, sleep disruption, nutritional restriction, reduced activity, and systemic glucocorticoid exposure may all interfere with growth and skeletal development.

Long-term studies in children aged 6 months to 5 years have demonstrated sustained clinical benefit and an acceptable safety profile with dupilumab treatment [62]. A broader systematic review of pediatric evidence similarly found favorable efficacy and quality-of-life outcomes, without identifying osteoporosis or impaired skeletal development as established adverse effects [64]. However, follow-up durations remain short compared with the time required to assess peak bone mass or adult fracture outcomes.

More directly relevant evidence emerged from a 2026 post hoc analysis of a phase 3 randomized, placebo-controlled trial and its open-label extension. Dupilumab treatment was associated with improvements in linear growth measures and favorable changes in bone-related biomarkers among children with AD [59]. These findings support the possibility that controlling severe type 2 inflammation and reducing its systemic consequences may facilitate recovery of normal growth and bone formation.

Nevertheless, several qualifications are essential. The analysis was post hoc, bone outcomes were not the primary endpoints of the original trial, and biochemical markers are not equivalent to DXA-measured bone mass, bone microarchitecture, or fracture reduction. Changes in growth and biomarkers may reflect improved disease control, nutrition, sleep, and physical activity; reduced glucocorticoid exposure; direct cytokine effects; or a combination of these mechanisms. The study therefore provides an encouraging clinical signal rather than definitive evidence of an osteoprotective effect.

### 6.5. Pharmacovigilance Signals and the Risk of Misinterpretation

Spontaneous reporting databases are valuable for detecting uncommon or unexpected adverse event signals after a medication enters widespread use. An analysis of the US Food and Drug Administration Adverse Event Reporting System identified established and potential adverse-event signals associated with dupilumab [63]. Such analyses can generate hypotheses but cannot determine incidence, relative risk, or causality.

Reports submitted to pharmacovigilance databases are influenced by stimulated reporting, duplicate entries, missing clinical information, selective documentation, and the absence of an appropriate untreated comparator [63]. They frequently lack information on baseline osteoporosis, previous fractures, cumulative glucocorticoid exposure, disease severity, age, menopausal status, nutritional status, falls, and concomitant medications [63]. Severe AD itself and its treatment history may also be associated with increased skeletal risk, creating substantial potential for confounding by indication [13,17,25,26,31].

A second individual observation was reported in [74]. A 45-year-old man with severe AD developed musculoskeletal discomfort and was found to have DXA-defined osteoporosis (T-score −2.8) after approximately 18 months of dupilumab treatment. The abstract reported normal calcium and vitamin D concentrations, elevated bone resorption markers, and no history of systemic corticosteroid use, thyroid disease, or other recognized secondary causes of osteoporosis. Although this case is noteworthy because several conventional risk factors were reportedly absent, a single post-treatment DXA measurement cannot establish that bone loss developed during therapy or that dupilumab was responsible. Information regarding pretreatment BMD, family history, BMI, gonadal status, smoking, alcohol consumption, physical activity, and other potentially occult secondary causes was unavailable or insufficiently detailed [74].

Therefore, an osteoporosis or fracture report occurring during dupilumab therapy should not automatically be interpreted as an adverse effect caused by IL-4/IL-13 blockade [31,58,63]. Temporal coexistence is insufficient to distinguish drug toxicity from background disease risk, pre-existing skeletal vulnerability, previous systemic glucocorticoid exposure, or an unrelated traumatic event [17,25,26,31,63]. A pharmacovigilance signal should be regarded as hypothesis-generating and requires confirmation through comparative cohort studies or prospectively designed skeletal safety investigations [63].

### 6.6. Other Biologics and Janus Kinase Inhibitors

Other targeted treatments for AD include biologics directed against IL-13 and oral Janus kinase (JAK) inhibitors. Current guidelines strongly recommend several of these agents for appropriately selected adults with moderate-to-severe disease [60,61]. Their effects on bone health remain poorly characterized because skeletal endpoints have rarely been incorporated into AD clinical trials.

Selective IL-13 inhibition raises mechanistic questions similar to those surrounding dupilumab, but no consistent clinical signal currently indicates increased risk of osteoporosis or fractures. JAK inhibitors influence multiple cytokine pathways and may, in theory, modify immune–bone interactions more broadly. However, their clinical safety assessment has focused primarily on infections, laboratory abnormalities, thromboembolic events, cardiovascular outcomes, and malignancy rather than on longitudinal skeletal health.

Differences among targeted therapies should not be inferred exclusively from their molecular targets. The net bone effect may depend on the magnitude of disease control, reduction in glucocorticoid use, improvement in activity and nutrition, age, baseline fracture risk, and treatment duration. Direct comparative studies with standardized bone outcomes are required before a hierarchy of skeletal safety or benefit can be proposed.

The potential direct and indirect skeletal implications of systemic therapies used for moderate-to-severe AD are summarized in Table 2.

Overall, systemic glucocorticoids remain the treatment class with the most clearly established adverse skeletal effects, whereas the bone consequences of conventional immunosuppressants, biologics, and JAK inhibitors remain insufficiently characterized. Targeted therapies may indirectly improve skeletal health through sustained disease control and glucocorticoid avoidance, but current evidence does not justify assigning them either osteoprotective or osteotoxic properties.

### 6.7. Therapeutic Selection in Patients with Elevated Skeletal Risk

The presence of osteoporosis or increased fracture risk should encourage individualized systemic treatment rather than therapeutic undertreatment of AD. Persistently uncontrolled disease may perpetuate sleep disturbance, inactivity, restrictive eating, systemic inflammation, and repeated glucocorticoid rescue courses. Effective steroid-sparing treatment may therefore be part of a broader bone-risk reduction strategy.

For patients with previous fragility fractures, low BMD, prolonged glucocorticoid exposure, impaired growth, or multiple conventional risk factors, baseline skeletal evaluation should be determined by age and absolute risk rather than by dupilumab exposure alone. Existing osteoporosis should be managed according to established bone-health recommendations, without assuming that control of AD will be sufficient to reverse skeletal disease.

Available evidence does not justify additional DXA monitoring solely because dupilumab has been initiated. Monitoring should instead be guided by conventional indications for osteoporosis, systemic glucocorticoid exposure, life stage, previous fractures, and the cumulative risk profile described in Section 5. Conversely, an incident low-trauma fracture during targeted therapy warrants clinical evaluation but should not be attributed automatically to the medication.

### 6.8. Research Priorities

Future trials of systemic AD therapies should prospectively incorporate skeletal outcomes in selected high-risk populations. Pediatric studies should assess height velocity, pubertal stage, nutritional status, body composition, bone-age–appropriate DXA measurements, and relevant bone-turnover markers. Adult studies should include baseline and longitudinal DXA, vertebral fracture assessment, trabecular bone score where available, muscle function, falls, and adjudicated low-trauma fractures.

Analyses should document cumulative systemic glucocorticoid exposure before and during targeted treatment. They should also account for disease severity, physical activity, BMI, vitamin D status, dietary restriction, smoking, menopause, and concomitant medications. Comparisons among untreated active disease, conventional systemic therapy, biologics, and JAK inhibitors would help distinguish the skeletal effects attributable to disease control from those attributable to a particular molecular intervention.

At present, the most defensible interpretation is that effective targeted treatment may reduce several indirect determinants of bone fragility and facilitate avoidance of glucocorticoids. Dupilumab has not been demonstrated to cause osteoporosis, and emerging pediatric findings suggest potential improvement in growth and bone-related biomarkers [59]. These observations remain insufficient, however, to establish a direct osteoprotective effect or to replace conventional fracture-risk assessment and osteoporosis management.

Overall, currently available evidence does not establish either a protective or a detrimental effect of dupilumab on fracture incidence in patients with AD. Although improvements in disease control, reduced systemic glucocorticoid exposure, and favorable changes in pediatric growth-related biomarkers may suggest potential indirect skeletal benefits, these observations cannot be considered evidence of fracture prevention [58,59]. Conversely, isolated reports of osteoporosis during dupilumab treatment do not establish treatment-related bone toxicity. Prospective longitudinal studies incorporating baseline and follow-up DXA measurements, standardized bone turnover markers, appropriate comparator groups, and adjudicated fragility fractures are needed to determine the long-term skeletal effects of dupilumab and other targeted therapies.

## 7. Risk-Adapted Screening and Clinical Assessment

The available evidence does not support universal osteoporosis screening solely on the basis of an AD diagnosis. Although meta-analyses indicate modestly increased risks of osteoporosis and fractures, absolute risk is heterogeneous and depends substantially on age, sex, disease severity, systemic glucocorticoid exposure, nutritional status, body composition, physical activity, comorbidities, and previous fractures [13,31]. Screening should therefore follow a risk-adapted model that integrates AD-related exposures with established osteoporosis recommendations [65,66,69].

### 7.1. Principles of Risk-Adapted Assessment

The objective of screening is not merely to identify a low DXA value but to detect individuals in whom skeletal assessment could alter clinical management. AD should be viewed as a potential risk-enhancing condition rather than as an independent indication for DXA in every patient.

A practical assessment begins with three questions:

- Has the patient already experienced a fragility fracture?
- Does the patient meet established age- or sex-based indications for osteoporosis screening?
- Are AD-related or treatment-related factors sufficiently important to justify earlier investigation?

A history of hip or vertebral fragility fracture establishes a high-risk clinical phenotype irrespective of whether BMD is in the osteoporotic range. Other fractures occurring after minimal trauma should prompt evaluation for osteoporosis and secondary causes, particularly in adults aged ≥50 years [65,66]. Vertebral fractures may remain clinically silent; height loss, new kyphosis, or persistent thoracic or lumbar pain should therefore raise suspicion even without a documented traumatic event.

AD-specific assessment should include disease onset, duration, severity, flare frequency, body-surface involvement, sleep disturbance, physical limitations, dietary restrictions, and history of systemic therapy. Particular attention should be paid to cumulative oral or parenteral glucocorticoid exposure, including repeated short courses that patients may not recognize as long-term treatment.

### 7.2. Initial Clinical Risk Evaluation

A structured history should document conventional osteoporosis and fracture-risk factors: previous low-trauma fracture; parental history of hip fracture; menopause and age at menopause; hypogonadism or menstrual disturbance; low BMI, weight loss, or impaired growth; smoking and excessive alcohol consumption; reduced mobility or physical inactivity; recurrent falls or impaired balance; low calcium or protein intake; vitamin D deficiency or limited sunlight exposure; malabsorption or restrictive diets; chronic kidney, liver, endocrine, gastrointestinal, or inflammatory disease; medications that impair bone or increase fall risk; systemic glucocorticoid dose, duration, pattern, and cumulative exposure.

For patients with AD, additional questions should address food avoidance, nocturnal pruritus, daytime fatigue, sedating medication use, avoidance of exercise because of sweating or embarrassment, and work patterns that disrupt sleep or regular meals. Shift work does not currently represent an independent indication for DXA, but it may strengthen concern when combined with sleep disruption, low activity, nutritional irregularity, falls, and other established risk factors.

Physical examination may identify height loss, kyphosis, low body mass, muscle weakness, impaired balance, gait instability, signs of endocrine disease, or delayed growth and puberty. Height should be compared with previous measurements whenever possible. In children and adolescents, a longitudinal growth trajectory is more informative than a single measurement.

### 7.3. Who Should Undergo DXA?

General population recommendations remain the foundation of screening. Current guidance supports DXA screening in women aged ≥65 years and in younger postmenopausal women whose clinical risk assessment indicates increased fracture risk [65,66,69]. Some professional guidelines also recommend testing men aged ≥70 years and younger men with significant risk factors [65,66]. The 2025 US Preventive Services Task Force concluded that evidence remains insufficient to determine the balance of benefits and harms of population screening in men, emphasizing the distinction between the absence of evidence for universal screening and the need for individualized testing in high-risk clinical settings [69].

AD itself should not automatically lower these thresholds. Earlier DXA may nevertheless be appropriate when AD coexists with one or more major skeletal-risk determinants, particularly: previous fragility fracture; repeated or prolonged systemic glucocorticoid exposure; severe, persistent, or long-standing disease accompanied by additional risk factors; early menopause or hypogonadism; low BMI or substantial weight loss; prolonged nutritional restriction or malabsorption; impaired growth or delayed puberty; recurrent falls, sarcopenia, or reduced mobility; radiographic osteopenia or suspected vertebral compression; a disorder or medication independently associated with secondary osteoporosis.

For adults initiating or continuing systemic glucocorticoid therapy at a prednisone-equivalent dose of ≥2.5 mg/day for more than 3 months, early fracture risk and BMD assessment should follow the glucocorticoid-induced osteoporosis recommendations [48,50]. Risk may also be clinically meaningful after recurrent high-dose courses, even when continuous exposure does not exceed three months. Dose history should therefore be evaluated cumulatively rather than through a simple current-use variable.

There is no evidence supporting baseline or serial DXA solely because dupilumab or another targeted AD therapy is initiated. Patients receiving these agents should undergo skeletal assessment based on their age, fracture history, prior glucocorticoid exposure, and overall risk profile.

### 7.4. DXA Acquisition and Interpretation in Adults

Central DXA of the lumbar spine and hip remains the standard method for measuring areal BMD. In postmenopausal women and men aged ≥50 years, T-scores are used for diagnostic classification. A T-score of −2.5 or lower at an accepted skeletal site is consistent with osteoporosis, while values between −1.0 and −2.5 indicate low bone mass. However, fracture risk exists along a continuum, and many fragility fractures occur in individuals whose BMD does not reach the osteoporotic threshold [65,66,67].

In premenopausal women and men younger than 50 years, Z-scores are generally preferred. A Z-score of −2.0 or lower is described as “below the expected range for age,” whereas a value above −2.0 is “within the expected range for age” [67]. Osteoporosis should not be diagnosed in younger adults from BMD alone without considering fragility fractures, secondary causes, and the clinical context.

Interpretation may be affected by degenerative spinal changes, vertebral deformity, vascular calcification, previous surgery, positioning, and differences between DXA devices. Apparent preservation of lumbar BMD in an older patient does not exclude osteoporosis when degenerative disease artificially elevates the measurement. Hip values, vertebral imaging, and clinical fracture history may provide more reliable information in such circumstances.

The observation that older patients with AD may experience vertebral fractures despite similar BMD to controls suggests that DXA does not capture all dimensions of skeletal strength [24]. Bone microarchitecture, falls, muscle function, and cumulative exposure may contribute independently. A normal or mildly reduced BMD should therefore not end the evaluation of a patient with a convincing fragility-fracture phenotype.

### 7.5. Pediatric and Adolescent Assessment

Routine DXA screening is not justified for all children with AD. Most childhood fractures occur during normal physical activity or after trauma, and their interpretation differs fundamentally from that of fragility fractures in older adults [18,20,75]. Investigation should be considered when AD coexists with clinically significant risk factors, such as recurrent low-trauma fractures, vertebral compression fractures, impaired growth, delayed puberty, low body weight, prolonged nutritional restriction, chronic systemic glucocorticoid therapy, reduced mobility, or another disorder or treatment affecting bone metabolism [48,75].

In children and adolescents, Z-scores—not T-scores—should be used. DXA results must be interpreted in relation to age, sex, body size, pubertal stage, and growth [75]. Short stature may artifactually lower areal BMD because DXA is a two-dimensional technique and smaller bones contain less mineral within the projected area. Height-adjusted measures or bone mineral apparent density may therefore be required. The preferred skeletal sites generally include the posterior–anterior lumbar spine and the total body, less the head, depending on the clinical circumstances, the availability of appropriate reference data, and local pediatric expertise [75].

A low pediatric BMD value alone is insufficient to diagnose osteoporosis. A low-trauma vertebral compression fracture is highly significant, whereas the number, anatomical location, and trauma mechanism of long-bone fractures must be evaluated alongside BMD and the underlying clinical condition. Referral to a pediatric bone specialist is appropriate when interpretation is uncertain, a primary or secondary bone disorder is suspected, or pharmacological treatment is being considered [75].

The emerging observation that dupilumab may improve growth and bone-related biomarkers in children with moderate-to-severe AD is encouraging but does not eliminate the need to investigate persistent growth failure, recurrent fractures, or substantial glucocorticoid exposure [59]. Improvement in skin disease should not be assumed to automatically normalize skeletal development.

### 7.6. Vertebral Fracture Assessment and Additional Imaging

Vertebral fracture assessment performed with DXA or conventional lateral spinal radiography may identify previously unrecognized vertebral compression fractures. It should be considered when clinical features raise suspicion, including: historical height loss or progressive loss of stature; unexplained back pain; kyphosis; systemic glucocorticoid exposure; low BMD combined with advanced age; a previous nonvertebral fragility fracture.

The presence of a vertebral fracture may substantially change risk classification and treatment decisions, even when BMD is not in the osteoporotic range [65,66]. This is especially relevant to older patients with AD because preliminary evidence suggests that vertebral fractures may be disproportionately represented [24,31].

In selected adults, trabecular bone score may offer additional insight into lumbar-spine texture and fracture risk, but it should not replace BMD or clinical assessment. Quantitative computed tomography and other imaging modalities may be useful in specialized circumstances but are not appropriate for routine AD-related screening.

### 7.7. Laboratory Evaluation for Secondary Causes

Laboratory testing should be guided by clinical context rather than performed indiscriminately in every patient with AD. In individuals with low BMD, fragility fractures, impaired growth, or substantial glucocorticoid exposure, an initial evaluation may include: complete blood count; serum calcium, phosphate, creatinine, and alkaline phosphatase; liver-function tests; serum 25-hydroxyvitamin D; thyroid-stimulating hormone; parathyroid hormone when calcium or vitamin D abnormalities are present; additional tests for malabsorption, hypogonadism, inflammatory disease, or monoclonal gammopathy when clinically indicated.

In pediatric patients, calcium, phosphate, alkaline phosphatase, vitamin D status, renal function, pubertal development, and dietary intake require age-appropriate interpretation. Bone-turnover markers may be difficult to interpret during growth because physiological remodeling varies substantially with age and puberty.

Bone-turnover markers can provide information about remodeling and treatment response in selected adults but are not diagnostic of osteoporosis. Likewise, serum RANKL, OPG, IL-4, IL-13, IL-31, and IL-33 should currently be regarded as research biomarkers. Their measurement is not standardized for routine clinical decision-making in patients with AD [14,36,46].

### 7.8. Fracture-Risk Tools and Their Limitations

FRAX estimates the 10-year probability of hip and major osteoporotic fractures using clinical risk factors, with or without femoral-neck BMD. It can support treatment decisions in age-appropriate adults but has several limitations in the AD population. AD is not included as a specific variable, and the tool does not directly account for disease severity, pruritus, sleep disruption, nutritional restriction, recurrent falls, or intermittent courses of glucocorticoids.

FRAX includes glucocorticoid exposure as a dichotomous variable, although dose adjustment can be applied in some circumstances. It may underestimate risk in patients receiving high doses, repeated courses, or prolonged therapy and in those with frequent falls, multiple recent fractures, or substantial frailty [48,50,65,66]. The calculated probability should therefore support—not replace—clinical judgment.

FRAX is not validated for children or adolescents and should not be applied to pediatric AD. Its role is also limited in younger adults, in whom absolute 10-year fracture probabilities may remain low despite clinically important secondary bone disease.

### 7.9. Follow-Up DXA and Longitudinal Monitoring

Repeat DXA should be performed only when the result is expected to influence management. Fixed annual testing is unnecessary for every patient with AD. Follow-up intervals should be individualized according to baseline BMD, age, fracture risk, systemic glucocorticoid exposure, treatment initiation or change, and the anticipated rate of bone loss [68].

When repeat testing is indicated, comparison should ideally be performed using the same DXA facility and device. Changes should be interpreted using the facility-specific least significant change rather than assuming any numerical difference is biologically meaningful [67,68]. Small changes may reflect measurement variability, positioning, differences in analysis, or artifacts.

Shorter monitoring intervals may be appropriate in circumstances associated with rapid skeletal change, including high-dose systemic glucocorticoid therapy, major weight loss, newly diagnosed malabsorption, or initiation of osteoporosis treatment. Longer intervals are reasonable when BMD is stable, risk factors are unchanged, and no new fracture has occurred.

An incident fragility fracture, substantial height loss, new systemic glucocorticoid exposure, or major change in clinical risk should trigger reassessment irrespective of the planned DXA interval. Changes in AD treatment alone do not mandate repeat DXA unless they materially alter the patient’s overall skeletal-risk profile.

### 7.10. A Proposed Clinical Algorithm for Patients with AD

Importantly, the proposed algorithm represents an evidence-informed clinical framework developed by the authors rather than a validated AD-specific screening tool. Its foundation consists of established osteoporosis guidelines addressing age- and sex-based screening, previous fragility fractures, glucocorticoid-induced osteoporosis, and age-appropriate DXA interpretation [48,50,65,66,67,68,69]. By contrast, the incorporation of AD severity, disease duration, cumulative inflammatory burden, and associated lifestyle factors as additional risk modifiers reflects expert interpretation of the available observational and mechanistic evidence. No prospectively validated algorithm or AD-specific DXA threshold is currently available. Accordingly, the proposed pathway should support individualized clinical judgment rather than replace established osteoporosis screening recommendations.

A practical risk-adapted pathway can be organized into four stages.

Stage 1: Identify established screening indications. Patients should first be assessed according to age-, sex-, and guideline-based recommendations for osteoporosis. Previous hip or vertebral fragility fracture, known osteoporosis, or another major secondary cause should prompt direct evaluation and management [65,66,69].

Stage 2: Identify AD-related risk enhancers. These include severe or long-standing disease, systemic glucocorticoid exposure, low BMI, nutritional restriction, vitamin D deficiency, impaired growth, reduced activity, sleep disturbance, falls, sarcopenia, and occupational schedules that aggravate these factors.

Stage 3: Select age-appropriate investigations. Adults at increased risk may require DXA of the hip and lumbar spine, fracture-risk assessment, vertebral imaging, and laboratory evaluation for secondary causes. Children require growth- and size-adjusted assessment, with specialist interpretation when DXA is performed.

Stage 4: Intervene according to absolute risk. Management may include minimizing systemic glucocorticoid exposure, optimizing AD control, correcting nutritional deficiencies, promoting weight-bearing and resistance activities, reducing fall risk, addressing sleep and occupational factors, and initiating osteoporosis treatment when conventional criteria are met.

This model avoids two opposite errors: indiscriminate DXA testing of every patient with AD and failure to recognize high-risk individuals because AD is considered exclusively a cutaneous disease. The purpose of risk-adapted screening is not to redefine AD as a direct cause of osteoporosis, but to identify combinations of inflammatory, therapeutic, lifestyle, occupational, and conventional factors that make clinically significant skeletal fragility more likely.

**Figure 2:**
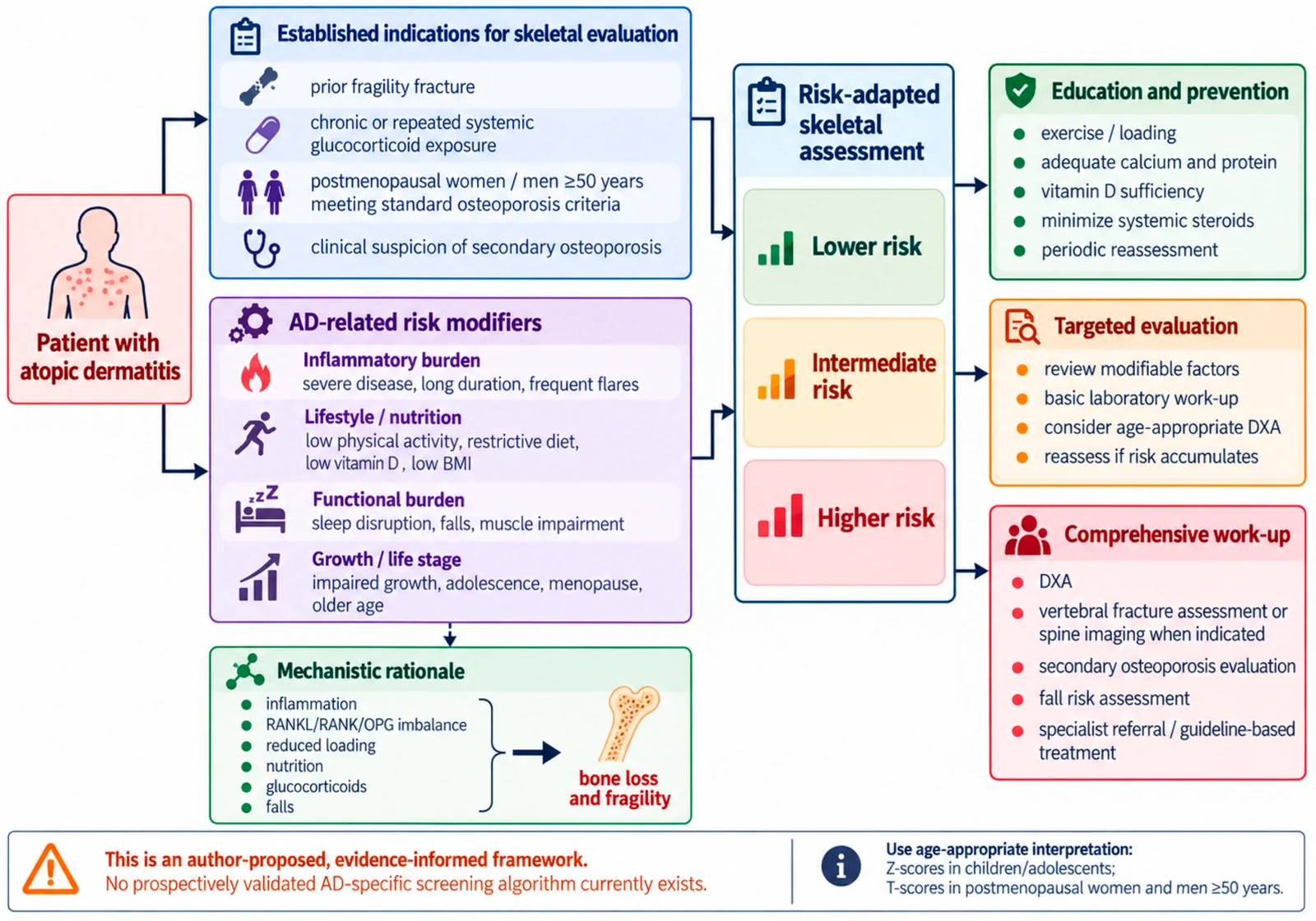
**Mechanistic-clinical framework for skeletal assessment in atopic dermatitis.** This figure presents an author-proposed, evidence-informed framework for skeletal assessment in patients with atopic dermatitis. The approach integrates established indications for bone health evaluation—such as prior fragility fracture, chronic or repeated systemic glucocorticoid exposure, postmenopausal status or men aged ≥50 years meeting standard osteoporosis criteria, and clinical suspicion of secondary osteoporosis—with AD-related risk modifiers, including inflammatory burden, nutritional and lifestyle factors, functional burden, and life-stage or growth-related considerations. These clinical modifiers are supported by a mechanistic rationale involving inflammation, RANKL/RANK/OPG imbalance, reduced loading, nutritional compromise, glucocorticoid exposure, and fall risk, all of which may contribute to bone loss and skeletal fragility. Based on the combined risk profile, patients may be stratified into lower-, intermediate-, or higher-risk groups to guide management ranging from education and prevention, to targeted evaluation, to comprehensive work-up. This framework is intended to support individualized clinical judgment and does not represent a prospectively validated AD-specific screening algorithm. Age-appropriate interpretation of bone mineral density remains essential, including Z-scores in children and adolescents and T-scores in postmenopausal women and men aged ≥50 years.

The proposed risk-adapted pathway for skeletal assessment and management in patients with AD is illustrated in Figure 2.

## 8. Discussion

### 8.1. Principal Findings

This review indicates that AD is associated with a modest increase in osteoporosis and fracture risk, but the relationship is heterogeneous, multifactorial, and not necessarily causal. The most recent cohort-based meta-analysis reported approximately 56% higher odds of osteoporosis and 8% higher odds of any fracture among individuals with AD [31]. The difference between these effect estimates is clinically and methodologically important. Osteoporosis diagnoses may be influenced by selective DXA testing and healthcare utilization, whereas fractures are more objective outcomes but include events caused by trauma, falls, and behavioral exposure rather than skeletal fragility alone.

Earlier meta-analytic evidence similarly identified increased risks of low BMD, osteopenia, osteoporosis, and fractures [13]. Nevertheless, individual studies have produced inconsistent results. Some large population-based cohorts reported modestly higher fracture rates, particularly in patients with severe disease [15,17,18,20,27,30], whereas studies incorporating direct BMD assessment have not consistently demonstrated lower BMD in patients with AD [16,24]. These findings suggest that fracture susceptibility may not be explained exclusively by reduced areal BMD.

The overall evidence supports five principal conclusions. First, the skeletal risk associated with AD is probably concentrated in susceptible subgroups rather than distributed uniformly across all patients. Second, disease severity and duration appear to be relevant modifiers, although their measurement remains inconsistent. Third, systemic glucocorticoid exposure is an important but incomplete explanation. Fourth, osteoimmunological pathways provide biological plausibility but do not yet establish a direct cytokine-mediated causal mechanism. Finally, current evidence favors risk-adapted clinical assessment over universal DXA screening based solely on AD.

### 8.2. Interpreting the Association Between AD and Bone Fragility

The distinction between association and causation is central to interpreting the literature. Patients with AD differ from unaffected comparators in numerous characteristics that may independently influence bone and fracture risk. These include systemic glucocorticoid exposure, lower physical activity, sleep disruption, dietary restriction, altered body composition, vitamin D deficiency, psychological comorbidity, sedating medication use, and increased healthcare contact.

Residual confounding is therefore likely even in well-designed cohort studies. Administrative datasets frequently lack standardized measures of AD activity, lifetime treatment exposure, diet, physical activity, falls, menopause, muscle function, and trauma mechanism. Severe disease is commonly defined through prescriptions or specialist care, meaning that apparent severity gradients may partly reflect treatment intensity or healthcare utilization.

Surveillance bias may also contribute to the association with diagnosed osteoporosis. Patients with chronic inflammatory disease or systemic glucocorticoid exposure may be more likely to undergo DXA and receive an osteoporosis code. Conversely, osteoporosis may remain undetected in comparators who do not undergo screening. Fractures are less sensitive to this form of bias, but coding-based studies may not distinguish low-trauma fragility fractures from sports injuries, occupational trauma, or accidental childhood fractures.

The substantial heterogeneity reported in meta-analyses supports this interpretation. In the 2026 analysis, heterogeneity reached 99.9% for osteoporosis and 82.1% for overall fractures [31]. A pooled association obtained under such conditions should be interpreted as an average across clinically dissimilar populations rather than as a uniform effect applicable to every patient with AD.

Nevertheless, heterogeneity does not invalidate the association. Similar findings across different countries, age groups, databases, and study designs suggest that the signal is unlikely to be explained entirely by chance [12,13,23,31]. The key question is therefore not whether AD is statistically associated with skeletal outcomes, but which combination of disease, treatment, and patient characteristics yields a clinically meaningful absolute risk.

### 8.3. BMD and Fracture Risk Are Related but Not Interchangeable

The discordance between BMD and fracture findings is one of the most informative features of the current evidence. Young adults with AD did not demonstrate a significantly higher overall prevalence of low BMD, although earlier disease onset, longer duration, and lower BMI were associated with less favorable measurements within the AD population [16]. Similarly, a study of older adults reported comparable BMD after matching but a higher risk of vertebral fractures, although estimates were based on few events and had wide confidence intervals [24].

Several explanations are possible. First, DXA measures areal BMD but does not fully capture trabecular microarchitecture, cortical porosity, bone-material properties, or remodeling dynamics. Second, fracture risk reflects both skeletal strength and the probability of trauma or falling. Sleep deprivation, sedating medications, impaired balance, muscle weakness, and occupational hazards could increase fractures without materially reducing BMD. Third, lumbar BMD may be artificially elevated in older adults by degenerative changes, potentially obscuring underlying skeletal fragility.

Fracture location is also relevant. The relatively consistent signals for vertebral and lower-limb fractures require confirmation, as these outcomes may arise from different mechanisms [24,31]. Vertebral fractures are more suggestive of skeletal fragility but can remain undiagnosed, whereas lower-limb fractures may be influenced more strongly by falls, physical activity, and trauma exposure.

Future studies should therefore avoid treating osteoporosis codes, low BMD, and fractures as equivalent outcomes. Combining DXA with vertebral imaging, trabecular bone score, muscle assessment, falls, and adjudicated trauma mechanism would provide a more complete skeletal phenotype.

### 8.4. Disease Activity, Glucocorticoids, and Lifestyle: Competing or Complementary Explanations?

Systemic glucocorticoids have an established adverse effect on bone and represent the most clinically actionable contributor to skeletal risk [48,49,50]. Their continued use in AD is concerning because repeated short courses can generate substantial cumulative exposure [25,26]. However, studies that adjust for oral glucocorticoids suggest that they account for only part of the observed association [17].

This residual risk should not automatically be attributed to systemic inflammation. Glucocorticoid exposure is difficult to quantify accurately, particularly when prescriptions occur across different healthcare settings or before entry into a database. Prescription records do not confirm adherence, whereas current-use variables may fail to capture cumulative prior exposure. Confounding by indication also remains important because patients receiving glucocorticoids generally have more severe disease.

Disease severity, treatment, and lifestyle should therefore be considered complementary rather than mutually exclusive explanations. Severe AD may increase inflammatory burden while simultaneously worsening sleep, reducing activity, promoting dietary restriction, and increasing the likelihood of systemic rescue treatment. These pathways can interact and reinforce one another over time.

Topical corticosteroids should be distinguished clearly from systemic exposure. Pediatric cohort data and controlled adult evidence have not demonstrated a clinically important adverse skeletal effect from the appropriate use of topical treatment [19,21]. Excessive concern regarding topical corticosteroids may produce undertreatment, persistent inflammation, sleep loss, and eventual escalation to systemic therapy. The clinically appropriate strategy is effective topical disease control with attention to potency, treated surface area, duration, and patient age—not avoidance based on an assumed equivalence with oral glucocorticoids.

Lifestyle factors further complicate causal interpretation. Lower exercise capacity has been reported in adolescents with AD, but whether this translates into impaired peak bone mass is unknown [55]. Vitamin D concentrations may be lower in more severe disease, yet supplementation trials have primarily assessed dermatitis severity rather than bone outcomes [51,52,53]. Similarly, sleep disturbance is common and clinically important, but its direct contribution to fracture risk in AD has not been quantified [57].

Shift work and occupational stress may amplify sleep, dietary, and physical activity disturbances, particularly among patients working rotating three-shift schedules. However, no current evidence demonstrates an AD-specific interaction between shift work and osteoporosis. These exposures are best interpreted as contextual modifiers within a cumulative risk model.

### 8.5. The Osteoimmunological and Therapeutic Paradox

The RANK–RANKL–OPG system provides a biologically plausible interface between chronic inflammation and bone remodeling [33,35,37,43]. The reported correlation between AD severity and the serum RANKL/OPG ratio, particularly in older women, is consistent with increased osteoclastogenic potential [14]. Nevertheless, circulating biomarker measurements cannot determine whether the signal originates from skin, bone, immune cells, or another tissue, nor whether it predicts subsequent bone loss or fractures.

The roles of IL-4 and IL-13 illustrate the hazards of linear mechanistic interpretation. Both cytokines can suppress osteoclastogenesis and promote anti-inflammatory macrophage phenotypes in experimental systems [38,39,42,44]. Yet excessive IL-4 signaling has enhanced osteoclast formation in a model of food-allergic enteropathy [47]. These apparently contradictory findings may reflect differences in concentration, exposure duration, tissue environment, cell-differentiation stage, and associated inflammatory signals.

The IL-33/IL-31 axis is similarly complex. IL-33 has demonstrated anti-osteoclastogenic effects in selected models, whereas IL-31 has been associated with low BMD and may connect type 2 inflammation with pruritus and sleep disruption [36,40,41]. Most of this evidence is preclinical or derived from conditions other than AD and should not be interpreted as confirmation of an AD-specific cytokine–fracture pathway.

This complexity is directly relevant to dupilumab. Because IL-4 and IL-13 can experimentally inhibit osteoclastogenesis, blockade of their shared receptor could theoretically remove an osteoprotective signal [38,39,42,44,46]. Conversely, effective treatment may reduce AD-related inflammation and barrier dysfunction, improve pruritus, sleep, quality of life, and physical functioning, and facilitate avoidance of systemic glucocorticoids [58,60,61,62,64]. The net clinical effect on bone cannot therefore be predicted from receptor pharmacology alone [37,43,46].

Available adult and pediatric safety data have not established osteoporosis or fractures as adverse effects of dupilumab [58,62,64]. Moreover, a 2026 post hoc pediatric analysis reported improved linear growth and favorable changes in bone-related biomarkers during treatment [59]. These findings argue against a simple model in which IL-4/IL-13 blockade necessarily damages bone.

However, the pediatric analysis does not prove that dupilumab increases BMD or prevents fractures. Biomarker and growth changes may result from improved overall health rather than from a direct effect on bone. Similarly, pharmacovigilance reports cannot establish causality because they lack appropriate denominators and detailed adjustment for baseline osteoporosis, disease severity, falls, and previous glucocorticoid exposure [63].

The two dupilumab-associated osteoporosis cases presented in [73,74] further illustrate this interpretative problem. Both observations generated a biologically plausible hypothesis based on the temporal relationship between treatment and the diagnosis of osteoporosis, but neither included a pretreatment DXA trajectory nor a sufficiently detailed baseline skeletal risk assessment. They should therefore stimulate prospective monitoring studies rather than support classification of osteoporosis as a demonstrated adverse effect of dupilumab.

The most defensible conclusion is therefore that dupilumab has not been shown to cause osteoporosis and may indirectly support skeletal health through disease control and glucocorticoid sparing. Evidence remains insufficient to classify it as directly osteoprotective.

### 8.6. A Lifespan Perspective

The clinical meaning of skeletal risk differs substantially across life stages. In childhood and adolescence, the primary concern is not conventional postmenopausal osteoporosis but impaired growth and failure to achieve optimal peak bone mass. Severe disease, restricted nutrition, low body weight, reduced activity, delayed puberty, and glucocorticoid exposure may have consequences that are not captured adequately by short-term fracture incidence.

Pediatric fracture studies have reported modest associations with AD [18,20], but childhood fractures are common and frequently traumatic. Without information on trauma mechanism, bone size, growth, and pubertal stage, increased fracture incidence cannot be assumed to represent osteoporosis. DXA interpretation also requires Z-scores and adjustment for growth and body size; applying adult T-score thresholds would be inappropriate.

In younger adults, low absolute fracture risk may obscure early biological effects. Disease duration, low BMI, nutritional deficiency, and previous glucocorticoid exposure may be more informative than chronological age alone [16]. Premenopausal women and men younger than 50 years require clinical interpretation based on Z-scores, secondary causes, and fracture history rather than BMD classification alone [67].

In older adults, AD-related factors are superimposed on menopause, age-related bone loss, sarcopenia, multimorbidity, polypharmacy, and falls. A modest relative risk may correspond to a clinically meaningful absolute increase in this population. Vertebral imaging and fall assessment may be particularly important when BMD appears relatively preserved, but clinical suspicion remains high [24].

### 8.7. Implications for Screening and Clinical Practice

No guideline currently recommends universal DXA screening for all patients with AD. The available association, although reproducible, is insufficiently specific and too heterogeneous to justify such an approach. Universal testing could increase costs, incidental findings, and inappropriate labeling, particularly in young individuals whose absolute fracture risk is low.

Conversely, ignoring bone health entirely may miss clinically important risk in patients with severe disease, systemic glucocorticoid exposure, low BMI, nutritional restriction, growth impairment, menopause, sarcopenia, falls, or previous fractures. The proposed algorithm in Figure 2 is intended to identify these higher-risk combinations.

Established osteoporosis recommendations should remain the foundation of clinical assessment [65,66,69]. AD functions as a potential risk enhancer that may justify closer evaluation when combined with conventional determinants. The strongest indication for early assessment remains a previous fragility fracture or clinically meaningful systemic glucocorticoid exposure [48,50].

DXA results should always be interpreted according to age and life stage [67]. A normal BMD does not exclude skeletal fragility in a patient with a vertebral or other low-trauma fracture, while a low areal BMD in a short child does not independently establish osteoporosis. Repeat testing should be individualized and performed only when the result is expected to influence management [68].

Clinical prevention should emphasize effective AD control, avoidance of unnecessary systemic glucocorticoids, adequate calcium and protein intake, correction of vitamin D deficiency, maintenance of healthy body weight, resistance and weight-bearing activity, sleep optimization, and fall prevention. These measures benefit general health even when a direct AD–bone causal pathway cannot be demonstrated.

### 8.8. Strengths and Limitations of the Review

A strength of this review is its integrated assessment of epidemiological, mechanistic, therapeutic, and diagnostic evidence published between January 2020 and July 2026. The review does not treat osteoporosis, BMD, and fractures as interchangeable outcomes and explicitly distinguishes direct clinical evidence from preclinical or extrapolated osteoimmunological data. It also incorporates a lifespan perspective and proposes a risk-adapted clinical framework rather than universal screening.

Particular attention was given to the therapeutic paradox surrounding IL-4/IL-13 inhibition. Emerging pediatric data were integrated without interpreting improvements in growth or bone biomarkers as evidence of fracture prevention [59]. Pharmacovigilance findings were treated as hypothesis-generating rather than causal evidence [63]. The meeting abstracts that motivated the review were similarly used to frame research questions and were not included as peer-reviewed evidence.

Several limitations must also be acknowledged. This was a critical narrative review with a structured search rather than a prospectively registered systematic review. Study selection, data extraction, and evidence synthesis were not performed by multiple independent reviewers, and a uniform risk-of-bias instrument was not applied. The search was centered on PubMed/MEDLINE, supplemented by reference screening and metadata verification, and may have missed eligible studies indexed exclusively in other databases.

Quantitative pooling was not repeated because recent meta-analyses were available [13,23,31]. Consequently, this review cannot determine whether alternative analytical decisions would change the pooled estimates. Publication bias and selective reporting remain possible, particularly for small biomarker or mechanistic studies.

The clinical literature was heterogeneous in age, AD definition, severity classification, treatment exposure, comparator selection, and outcome ascertainment. Many studies relied on administrative diagnoses and could not distinguish fragility fractures from traumatic fractures. Direct evidence regarding bone microarchitecture, muscle function, falls, and longitudinal BMD change was sparse.

Mechanistic interpretation was limited by reliance on experimental models, periodontal disease, allergic enteropathy, rheumatoid arthritis, and general osteoporosis research. These data establish biological plausibility but cannot confirm that identical pathways determine fracture risk in human AD. Finally, evidence concerning the skeletal effects of targeted therapy remains insufficient because bone outcomes were rarely prespecified in clinical trials. The meeting abstracts cited in this review were deliberately treated as hypotheses: [70] provided a preliminary cross-sectional clinical signal; [71,72] illustrated the coexistence of chronic disease activity, corticosteroid exposure, and reduced physical activity; and [73,74] raised questions regarding IL-4/IL-13 inhibition and bone remodeling.

Overall, this review supports recognition of bone fragility as a potentially relevant comorbidity in selected patients with AD, but it also demonstrates why the association should not be reduced to a single cytokine, treatment, or screening rule. The most appropriate clinical response is an individualized assessment based on cumulative absolute risk, while future research should determine whether AD-specific variables improve prediction beyond established determinants of osteoporosis.

## 9. Research Gaps and Future Directions

Despite increasing evidence of an association between atopic dermatitis and skeletal fragility, major uncertainties remain regarding causality, susceptible phenotypes, treatment effects, and optimal screening. Current evidence is dominated by retrospective cohorts, administrative databases, cross-sectional BMD studies, and meta-analyses of heterogeneous observational data [12,13,23,31]. The next stage of research should move beyond establishing whether an association exists and determine which patients are at clinically meaningful risk, through which pathways, and whether that risk can be modified.

### 9.1. Moving from Association to Causal Inference

The principal challenge is disentangling the effects of AD from those of systemic glucocorticoids, low BMI, nutritional restriction, reduced activity, sleep disturbance, comorbid allergic disease, socioeconomic factors, and healthcare utilization. Administrative databases can adjust only for variables that are recorded reliably, and disease severity is frequently inferred from prescriptions or healthcare encounters rather than measured directly.

Future epidemiological studies should use incident AD cohorts with active comparators, validated disease-severity measures, time-updated treatment exposure, and clearly defined skeletal outcomes. Analyses should distinguish low-trauma fractures from fractures caused by major trauma and should evaluate vertebral, hip, wrist, humeral, pelvic, and lower-limb fractures separately. Negative-control outcomes, propensity methods, marginal structural models, and mediation analyses could help clarify the respective contributions of disease activity and treatment.

Causal triangulation across different research designs would be particularly valuable. Concordant findings from longitudinal cohorts, mechanistic studies, treatment-response analyses, and genetically informed approaches would provide stronger evidence than any single design. At present, the available literature supports an association but does not demonstrate that AD-specific inflammation directly causes osteoporosis.

### 9.2. Standardized Definition of the Skeletal Phenotype

Bone health should not be represented by a single outcome. BMD, osteoporosis diagnoses, bone microarchitecture, vertebral deformities, bone turnover markers, muscle function, falls, and fractures capture distinct dimensions of skeletal fragility. Future studies should define these outcomes prospectively and report them separately.

A standardized skeletal assessment could include: lumbar-spine and hip DXA in adults; age-appropriate DXA sites and Z-scores in children; vertebral fracture assessment or spinal imaging; trabecular bone score where technically appropriate; serum bone-formation and resorption markers; height, weight, body composition, and muscle strength; falls and balance assessment; adjudicated low-trauma fractures.

Repeated measurement is essential. A cross-sectional difference in BMD cannot establish whether AD preceded bone loss, while a fracture outcome without baseline skeletal assessment cannot determine whether the event reflects reduced BMD, impaired bone quality, trauma exposure, or falls. Longitudinal designs should therefore combine imaging and clinical outcomes rather than relying exclusively on diagnostic codes.

### 9.3. Defining High-Risk AD Phenotypes

The modest pooled increase in fracture risk probably conceals substantial variation among patients [13,31]. Clinically important risk may be concentrated in distinct phenotypes rather than distributed uniformly across the AD population.

Potential high-risk profiles include: severe and persistent AD with extensive body-surface involvement; early-onset disease continuing through peak bone-mass acquisition; repeated or prolonged systemic glucocorticoid exposure; older women with long disease duration and elevated conventional risk; low BMI, restricted diets, or documented vitamin D deficiency; impaired growth or delayed puberty; reduced physical activity, sarcopenia, or recurrent falls; severe pruritus and chronic sleep disruption; multiple atopic diseases or other systemic inflammatory comorbidities; rotating shift work combined with circadian disruption and unfavorable lifestyle factors.

These candidate phenotypes require external validation. Studies should evaluate whether the combination of several moderate risk enhancers predicts skeletal outcomes more accurately than AD severity or glucocorticoid exposure alone.

### 9.4. Prospective Osteoimmunological Phenotyping

The RANK–RANKL–OPG system provides a plausible interface between cutaneous inflammation and bone remodeling, but human AD data remain limited [14,22]. Future studies should combine clinical disease measures with serum or tissue biomarkers and longitudinal skeletal outcomes.

Candidate panels may include RANKL, OPG, bone-specific alkaline phosphatase, procollagen type I N-terminal propeptide, C-terminal telopeptide, calcium, phosphate, parathyroid hormone, and 25-hydroxyvitamin D. Inflammatory profiling could include IL-4, IL-13, IL-31, IL-33, TNF-α, IL-6, and additional mediators reflecting the heterogeneity of chronic AD [33,34,35,36,37,38,39,40,41,42,43,44,45,46,47].

A major methodological requirement is temporal alignment. Biomarkers, AD severity, medication exposure, diet, physical activity, and bone-turnover measures should be collected at the same time points. Otherwise, correlations between isolated cytokine measurements and BMD may be difficult to interpret biologically. Laboratory methods, sample timing, fasting status, season, and preanalytical conditions should also be standardized.

These biomarkers should initially be evaluated as research tools rather than clinical screening tests. Demonstrating an association with AD severity is insufficient; a useful biomarker would need to predict longitudinal bone loss or fractures beyond conventional risk factors and produce clinically meaningful improvement in risk classification.

### 9.5. Pediatric Growth and Peak Bone-Mass Acquisition

Children and adolescents represent a priority population because skeletal consequences may emerge before conventional osteoporosis becomes detectable. Research should distinguish transient effects on growth from persistent failure to achieve genetic height potential or optimal peak bone mass.

Prospective pediatric cohorts should measure height velocity, pubertal development, dietary intake, physical activity, body composition, systemic glucocorticoid exposure, and age-adjusted skeletal parameters. DXA interpretation should account for short stature and delayed maturation, and fractures should be classified according to anatomical site, trauma mechanism, and recurrence.

The reported improvement in growth measures and bone-related biomarkers during dupilumab therapy supports a hypothesis for future investigation [59]. Confirmation will require prespecified skeletal endpoints, appropriate controls, longer follow-up, and assessment of whether early changes translate into improved peak bone mass. It will also be necessary to separate the effects of cytokine inhibition from those of improved sleep, nutrition, and activity, as well as reduced glucocorticoid exposure.

### 9.6. Comparative Skeletal Effects of Systemic Therapies

Current therapeutic evidence cannot determine whether biologics, JAK inhibitors, or conventional immunosuppressants differ in their long-term skeletal effects. Randomized trials are unlikely to be powered for fractures, but bone-focused substudies and prospective registries could address this gap.

Relevant comparisons should include: active uncontrolled disease versus effective systemic treatment; conventional immunosuppression versus targeted therapy; dupilumab versus selective IL-13 inhibition; biologics versus JAK inhibitors; patients with and without previous systemic glucocorticoid exposure; continuous targeted treatment versus discontinuation or treatment switching.

Outcomes should include changes in BMD, bone-turnover markers, body composition, muscle strength, falls, and fractures. Analyses must account for baseline differences because patients selected for particular therapies may vary in age, disease severity, comorbidity, and previous treatment exposure.

Pharmacovigilance analyses may identify hypotheses but should not be used in isolation to classify a therapy as osteotoxic [63]. Any skeletal signal requires confirmation with exposed and unexposed comparison groups, reliable denominator data, and adjustment for baseline risk.

### 9.7. Sleep, Circadian, and Occupational Dimensions

Sleep disturbance is a recognized burden of AD, but its role in skeletal outcomes remains largely unexplored [57]. Future studies should use validated sleep questionnaires, actigraphy, and objective activity measures to determine whether persistent nocturnal pruritus predicts reduced physical activity, impaired muscle function, falls, or bone loss.

Occupational variables should also be incorporated selectively. Shift schedule, cumulative night-work exposure, rotation speed, work intensity, heat exposure, protective clothing, opportunities for regular meals, and workplace access to skin care may influence both AD control and lifestyle-related skeletal risk. These variables are especially relevant in workers with severe disease, repeated systemic glucocorticoid treatment, or chronic sleep restriction.

The objective is not to establish shift work as an AD-specific cause of osteoporosis but to determine whether occupational circumstances amplify a cumulative risk profile. Such research could support practical interventions involving schedule adaptation, sleep optimization, regular nutrition, physical activity, and occupational-health surveillance.

### 9.8. Development and Validation of a Risk-Prediction Model

Existing fracture-risk tools do not include AD severity, pruritus, nutritional restriction, or intermittent glucocorticoid exposure. However, adding disease-specific variables is justified only if they improve prediction beyond established factors.

A proposed AD-specific model could initially evaluate: age and sex; menopausal or gonadal status; previous fragility fracture; BMI and body composition; AD duration and validated severity; cumulative systemic glucocorticoid dose; physical activity and muscle function; falls; dietary restriction and vitamin D status; sleep disturbance; conventional FRAX variables.

Model development should use sufficiently large prospective cohorts, penalized regression or other appropriate methods to reduce overfitting, and internal validation through bootstrapping or cross-validation. External validation in geographically and ethnically distinct populations would be essential. Calibration and clinical utility should be evaluated alongside discrimination.

A risk model should not be developed merely to produce a statistically significant score. It should identify patients whose management would change—for example, those who should undergo earlier DXA, vertebral imaging, laboratory evaluation, or fall-prevention assessment.

### 9.9. Digital Monitoring and Precision Prevention

Digital tools could improve the measurement of fluctuating AD severity and lifestyle exposures. Patient-reported symptom applications, wearable activity monitoring, sleep tracking, and electronic medication records may provide more accurate longitudinal exposure data than isolated clinic visits.

An integrated platform could combine AD activity, pruritus, sleep, physical activity, glucocorticoid use, nutritional indicators, falls, and BMD results. Such systems could identify periods of increased vulnerability, including severe flares accompanied by systemic treatment, inactivity, and sleep loss. Nevertheless, predictive algorithms must be validated prospectively and should not replace clinical assessment.

Precision prevention should remain clinically interpretable. Potential interventions include glucocorticoid avoidance, correction of nutritional deficiencies, maintenance of healthy body weight, resistance and weight-bearing activity, sleep optimization, fall prevention, and guideline-based osteoporosis therapy. The aim is to match the intensity of surveillance and prevention to absolute risk rather than to create unnecessary testing in patients with uncomplicated AD.

### 9.10. From Hypothesis-Generating Abstracts to Confirmatory Evidence

The meeting abstracts that motivated this review highlight a clinically plausible yet insufficiently studied relationship. Their principal value lies in identifying emerging observations, therapeutic paradoxes, and research questions. Because meeting abstracts frequently provide limited methodological detail and may not undergo the same review process as full publications, they should not be used to establish prevalence estimates, causal pathways, treatment-related harm, or screening recommendations.

Future full-length reports should provide clearly defined cohorts, comparator groups, AD severity, cumulative treatment exposure, age-appropriate bone assessment, adjustment for confounders, and transparent statistical methods. Prospective registration and publication of negative findings would reduce selective reporting and help determine whether preliminary signals are reproducible.

### 9.11. Translational Priorities

The immediate research priorities can be summarized as follows:

- Prospective lifespan cohorts combining standardized AD severity with repeated skeletal assessment.
- Precise quantification of cumulative systemic glucocorticoid exposure.
- Validation of high-risk clinical phenotypes rather than universal screening.
- Bone-focused studies of dupilumab, selective IL-13 inhibitors, and JAK inhibitors.
- Pediatric studies evaluating growth, puberty, and peak bone-mass acquisition.
- Integration of sleep, physical activity, nutrition, muscle function, falls, and occupational schedules.
- Validation of osteoimmunological biomarkers against longitudinal bone loss and fractures.
- Development and external validation of a clinically actionable risk-prediction model.

Addressing these priorities would shift the field from broad epidemiological association toward mechanistically informed and risk-adapted prevention. Until such evidence becomes available, clinical management should combine effective control of AD with established principles of bone health, avoidance of unnecessary systemic glucocorticoids, and targeted skeletal evaluation in patients with additional risk factors.

## 10. Conclusions

Current evidence supports a modest but clinically relevant association between AD and osteoporosis and fracture risk, particularly in patients with severe or long-standing disease and additional skeletal risk factors. This relationship is heterogeneous and cannot be attributed to a single mechanism. Chronic inflammation, systemic glucocorticoid exposure, nutritional restriction, low BMI, vitamin D deficiency, reduced physical activity, sleep disruption, muscle impairment, falls, and occupational or circadian stress may interact across the lifespan to influence bone strength. Osteoimmunological pathways involving the RANK–RANKL–OPG system and type 2 cytokines provide biological plausibility, but their net skeletal effects are context-dependent and remain incompletely validated in human AD. Available evidence does not demonstrate that dupilumab causes osteoporosis. On the contrary, emerging pediatric findings suggest potential improvements in linear growth and bone-related biomarkers, although these observations do not yet establish a direct osteoprotective effect or fracture prevention. AD alone is not sufficient to justify universal DXA screening. Skeletal assessment should be individualized based on age, prior fragility fractures, disease severity and duration, cumulative systemic glucocorticoid exposure, nutritional and hormonal status, physical activity, muscle function, falls, and established osteoporosis recommendations. Particular attention is warranted during growth, after prolonged or repeated systemic glucocorticoid treatment, and in older or otherwise vulnerable patients. Effective control of AD, avoidance of unnecessary systemic glucocorticoids, correction of nutritional deficiencies, maintenance of healthy body composition, regular weight-bearing and resistance activity, sleep optimization, and fall prevention constitute the most appropriate current preventive strategy. Prospective longitudinal studies integrating standardized AD phenotyping, treatment exposure, age-appropriate imaging, bone-turnover biomarkers, muscle assessment, and adjudicated fragility fractures are required to determine causality and validate AD-specific screening approaches.

## Figures and Tables

**Table 1:** Clinical and epidemiological studies evaluating the association between AD and bone fragility.

Study	Design and Population	Bone-Related Outcomes	Main Findings	Interpretation and Principal Limitations
Mukovozov et al., 2021 [12]	Systematic review of observational studies in children and adults with AD	BMD, osteopenia, osteoporosis, and fractures	Most included studies suggested an association between AD and impaired bone health, particularly in adults and patients with more severe disease, although findings were not uniform.	Supports an overall association but highlights substantial heterogeneity in populations, outcome definitions, disease severity, and adjustment for corticosteroid exposure.
Wu et al., 2021 [13]	Systematic review and meta-analysis of observational studies	Low BMD, osteopenia, osteoporosis, and fractures	AD was associated with osteoporosis (OR 1.95, 95% CI 1.18–3.23), osteopenia (OR 1.90, 95% CI 1.51–2.38), and fractures (OR 1.13, 95% CI 1.05–1.22).	Provides quantitative evidence of increased skeletal risk, but the estimates were derived predominantly from heterogeneous observational and cross-sectional studies.
Sakai et al., 2021 [14]	Cross-sectional biomarker study of patients with AD, with subgroup analyses according to age and sex	Serum RANKL, OPG, and RANKL/OPG ratio; history of osteoporosis or fracture	Serum RANKL levels and the RANKL/OPG ratio increased with AD severity. The association was most apparent among older women, and elevated ratios were observed in patients with osteoporosis or fractures.	Provides a mechanistic signal connecting AD severity with osteoclastogenic regulation, but the small, cross-sectional design precludes temporal or causal inference.
Lin et al., 2021 [15]	Nationwide matched retrospective cohort; 36,855 patients with AD and 147,420 individuals without AD	Incident fractures and anatomical fracture sites	Fractures occurred in 4.12% of patients with AD and 3.78% of controls. The 16-year cumulative incidence was 8.043% versus 7.366%, respectively. Severe AD was associated with higher fracture risk (HR 1.31, 95% CI 1.08–1.59).	Large population and long follow-up support a modest association, particularly in severe disease; claims-based diagnoses, residual confounding, and incomplete clinical characterization remain limitations.
Kim et al., 2021 [16]	Cross-sectional study of young Korean adults using population-based health and DXA data	Lumbar spine and femoral BMD; low BMD and osteoporosis	Overall low-BMD prevalence did not differ significantly between participants with and without AD. Within the AD group, earlier onset, longer disease duration, and lower BMI were associated with less favorable bone measurements.	Suggests that AD alone may not reduce BMD in young adults and that disease duration and constitutional factors may identify susceptible subgroups. The cross-sectional design limits causal interpretation.
Matthewman et al., 2022 [17]	Population-based matched cohort using UK primary-care data	Major osteoporotic fractures and fractures at specific anatomical sites; mediation by oral corticosteroids	Adults with atopic eczema had an increased risk of fractures, with stronger associations in more severe disease. Oral corticosteroid exposure explained only a limited proportion of the observed association.	Indicates that systemic corticosteroids are relevant but unlikely to account for the entire fracture signal. Residual confounding and outcome misclassification inherent to routine data are possible.
Ha et al., 2022 [18]	Nationwide pediatric cohort including 145,704 children	Incident fractures and associated clinical and social factors	Children with AD had a modestly higher fracture rate than children without AD (adjusted RR 1.08, 95% CI 1.05–1.10). The association was observed in both sexes.	The large sample supports a small pediatric association, but participants were ≤71 months old, fracture mechanisms were not fully characterized, and traumatic fractures could not always be separated from fragility fractures.
Imhof et al., 2022 [19]	Population-based pediatric cohort of patients with AD	Fractures in relation to topical corticosteroid exposure	Topical corticosteroid use was not associated with a clinically meaningful increase in pediatric fracture risk.	Reassuring regarding appropriately prescribed topical treatment; retrospective exposure assessment and limited information on adherence, treated body surface area, and cumulative absorption remain relevant.
Lee et al., 2023 [20]	Korean nationwide birth cohort with 1:3 propensity-score matching	Incident fractures after development of AD	Children who developed AD had a modest increase in subsequent fracture incidence compared with matched children without AD.	Temporal ordering strengthens the evidence, but administrative data cannot adequately distinguish bone fragility from fractures related to physical activity or accidental trauma.
Gether et al., 2023 [21]	Randomized controlled study in adults with AD comparing topical corticosteroid treatment with topical tacrolimus	Bone-homeostasis markers and metabolic outcomes	Short-term topical corticosteroid treatment did not produce a clinically important deterioration in the evaluated measures of bone homeostasis compared with tacrolimus.	Provides controlled evidence against a major short-term skeletal effect of topical corticosteroids; treatment duration and sample size were insufficient to evaluate osteoporosis or fracture outcomes.
Zhou et al., 2023 [23]	Meta-analysis and trial sequential analysis	Overall fracture risk	The pooled evidence supported an increased fracture risk in patients with AD, although the magnitude of the association was modest and the available studies remained heterogeneous.	Trial sequential analysis strengthened the assessment of the accumulated evidence, but conclusions remained dependent on observational studies and variable adjustment for confounders.
Hsiao et al., 2024 [24]	Retrospective hospital-based study of adults aged ≥45 years; 50 patients with AD and 386 controls undergoing DXA	BMD, FRAX estimates, and incident osteoporotic fractures	After propensity-score matching, BMD was similar between groups. AD was not significantly associated with fractures at all sites (HR 2.55, 95% CI 0.72–9.01), but vertebral fracture risk was increased (HR 6.80, 95% CI 1.77–26.17).	Suggests that fracture risk, particularly vertebral risk, may not be fully captured by BMD. The small number of fractures, short follow-up, and wide confidence intervals require cautious interpretation.
Jang et al., 2024 [25]	Nationwide longitudinal cohort of adults with AD evaluating long-term oral corticosteroid exposure	Osteoporosis, fractures, and multiple systemic safety outcomes	Longer cumulative oral corticosteroid use was associated with increasing risks of several adverse outcomes, including osteoporosis and fractures, compared with shorter exposure.	Supports cumulative systemic glucocorticoid exposure as a modifiable skeletal risk factor; confounding by indication and AD severity cannot be completely excluded.
Hagenström et al., 2024 [26]	Claims-data analysis of patients with AD receiving systemic glucocorticosteroids	Patterns of systemic glucocorticoid use and associated harms, including skeletal outcomes	Systemic glucocorticoids remained frequently used despite recommendations limiting their role, and greater exposure was associated with an increased burden of treatment-related adverse outcomes.	Demonstrates the real-world relevance of potentially avoidable glucocorticoid exposure, but claims data provide limited information on disease activity, adherence, and lifestyle confounders.
Matthewman et al., 2024 [27]	Seventy-one matched cohort analyses using UK primary-care records; up to 3.6 million people with eczema and 16.8 million comparators	Broad health outcomes, including osteoporosis and fractures	Atopic eczema was associated with multiple adverse health outcomes, including skeletal endpoints, with generally stronger associations in more severe disease.	An extremely large sample size and a consistent analytical framework facilitate comparisons across outcomes; multiplicity, coding accuracy, and residual confounding limit causal interpretation.
Lemeshow et al., 2025 [29]	Retrospective cohort study using the US Optum electronic health records database	Incident disease events, including osteoporosis and fractures	Adults with AD showed increased rates of several subsequent comorbidities, including bone-related outcomes, compared with adults without AD; risks varied according to disease and treatment characteristics.	Extends the association to a large contemporary US population, but outcome identification relied on electronic records and may be influenced by healthcare utilization and surveillance bias.
Chiesa Fuxench et al., 2025 [30]	Population-based UK adult cohort	Overall and site-specific incident fractures; analyses according to AD severity	Adults with AD had a modestly increased fracture risk, with stronger associations among patients with more severe disease and variation across anatomical fracture sites.	Supports a severity gradient but remains vulnerable to residual confounding by glucocorticoid exposure, BMI, physical activity, falls, and other osteoporosis determinants.
Liu et al., 2026 [31]	Meta-analysis of 10 cohort studies	Osteoporosis, any fracture, vertebral fracture, and lower-limb fracture	AD was associated with osteoporosis (OR 1.56, 95% CI 1.14–2.13), any fracture (OR 1.08, 95% CI 1.05–1.10), vertebral fracture (OR 1.14), and lower-limb fracture (OR 1.11). Heterogeneity was considerable for osteoporosis (I^2^ = 99.9%) and overall fractures (I^2^ = 82.1%).	Confirms a modest population-level association but shows that osteoporosis estimates vary markedly across studies; pooled results do not establish causality.
Peng et al., 2026 [32]	Prospective UK Biobank cohort of older adults with allergic diseases	Hospital-recorded osteoporosis and major osteoporotic fractures	Allergic disease burden was associated with subsequent skeletal outcomes, but associations differed between allergic phenotypes; the strongest signals were not consistently attributable to AD alone.	Provides prospective evidence for an allergy–bone relationship but should not be interpreted as AD-specific proof because of overlapping allergic conditions and hospital-recorded outcome ascertainment.
Mitroi et al., 2025 [70]	Twelve-month cross-sectional study; 120 adults aged 30–65 years with moderate-to-severe AD and 120 healthy controls	DXA-defined osteopenia and osteoporosis	Osteoporosis was reported in 25% of patients with AD versus 10% of controls, and osteopenia in 40% versus 22%. Longer disease duration, higher EASI scores, and systemic corticosteroid use were associated with less favorable outcomes.	Hypothesis-generating conference abstract without sufficient methodological detail or full peer-reviewed reporting; not included in the formal evidence base or quantitative synthesis.

**Abbreviations:** AD, atopic dermatitis; BMD, bone mineral density; CI, confidence interval; DXA, dual-energy X-ray absorptiometry; FRAX, Fracture Risk Assessment Tool; OPG, osteoprotegerin; OR, odds ratio; RANKL, receptor activator of nuclear factor-κB ligand; RR, risk ratio.

**Table 2:** Potential skeletal implications of systemic therapies used in atopic dermatitis.

Therapeutic Class or Agent	Principal Mechanism or Therapeutic Role	Potential Adverse Skeletal Pathways	Potential Protective or Indirect Skeletal Effects	Current Clinical Evidence and Practical Interpretation
Systemic glucocorticoids	Broad suppression of inflammatory and immune pathways; occasionally used for rapid control of severe flares	Reduced osteoblast differentiation and bone formation; osteoblast and osteocyte apoptosis; early increase in osteoclast survival; impaired calcium absorption; hypogonadism; muscle wasting and increased fall risk	Rapid suppression of severe inflammation, but benefits are generally transient and relapse may occur after withdrawal	The clearest treatment-related skeletal hazard in AD. Repeated courses and cumulative exposure may increase the risk of osteoporosis and fractures [25,26,48,49,50]. Current AD guidelines recommend against routine or prolonged use [60,61].
Cyclosporine	Calcineurin inhibition with suppression of T-cell activation; generally used for rapid, short-term control of severe AD	Experimental and transplant-related evidence suggests that high or prolonged exposure may increase bone turnover; renal dysfunction may indirectly disturb mineral metabolism	Rapid disease control may restore sleep and activity and reduce systemic glucocorticoid requirements	Direct AD-specific evidence regarding BMD and fractures is insufficient. Treatment is usually time-limited, and renal function and blood pressure require monitoring [60,61].
Methotrexate	Antimetabolite with immunomodulatory effects; conventional steroid-sparing systemic therapy	High oncological doses can impair bone formation; rare insufficiency fractures have been described in other treated populations, but relevance to low-dose AD therapy is uncertain	Steroid-sparing disease control may reduce glucocorticoid exposure and improve activity and quality of life	No convincing evidence demonstrates increased osteoporosis or fracture risk with standard low-dose treatment for AD. Skeletal monitoring should be guided by the patient’s underlying risk profile rather than by methotrexate exposure alone [60,61].
Azathioprine	Purine-antagonist immunosuppression affecting lymphocyte proliferation	No well-established direct osteotoxic mechanism at standard dermatological doses; long-term illness and concomitant glucocorticoids may confound observed skeletal outcomes	May provide steroid-sparing control in selected patients	AD-specific longitudinal BMD and fracture data are lacking. Bone surveillance should be guided by conventional risk factors and concomitant glucocorticoid exposure [60,61].
Mycophenolate mofetil	Inhibition of inosine monophosphate dehydrogenase and lymphocyte proliferation	Direct skeletal toxicity is not established; evidence from transplant populations is strongly confounded by glucocorticoids, organ dysfunction, and other immunosuppressants	Steroid-sparing control may reduce exposure to agents with clearer skeletal toxicity	No adequate AD-specific evidence supports either adverse or protective effects on BMD or fractures [60,61].
Dupilumab	IL-4 receptor-α blockade, inhibiting both IL-4 and IL-13 signaling	Theoretical concern that inhibition could remove anti-osteoclastogenic effects observed for IL-4 and IL-13 in selected experimental models; clinical relevance is unproven	Sustained disease control; reduced pruritus and sleep disruption; improved activity and nutrition; reduced systemic glucocorticoid requirements; possible improvement in pediatric growth and bone-formation biomarkers	Long-term adult and pediatric data have not established osteoporosis or fractures as treatment-related safety concerns [58,62,64]. A 2026 post hoc pediatric analysis reported improved linear growth and favorable changes in bone biomarkers [59], but no definitive effect on BMD or fractures has been demonstrated.
Selective IL-13 inhibitors	Selective neutralization of IL-13 signaling	IL-13 can suppress osteoclastogenic signaling and increase OPG expression experimentally; the consequences of long-term selective inhibition in humans remain uncertain	Effective disease control may improve sleep, activity, nutrition, and reduce systemic glucocorticoid exposure	No consistent clinical evidence currently links selective IL-13 inhibition with osteoporosis or fractures. Dedicated skeletal studies are unavailable; risk should not be inferred from cytokine biology alone [60,61].
JAK inhibitors	Intracellular inhibition of JAK-dependent signaling downstream of multiple cytokine receptors	Broad cytokine modulation could theoretically affect osteoclasts, osteoblasts, muscle, and immune–bone communication; treatment-specific clinical relevance remains unclear	Rapid disease and pruritus control may improve sleep, physical function, and glucocorticoid avoidance	AD trials have focused on infection, laboratory abnormalities, cardiovascular events, thrombosis, and malignancy rather than skeletal outcomes. No AD-specific hierarchy of osteoporosis or fracture risk can currently be established [60,61].
Phototherapy	Ultraviolet-mediated local and systemic immunomodulation; nonpharmacological option for selected patients	Repeated ultraviolet exposure has important cutaneous limitations, but direct adverse effects on bone are not expected; inconvenience may reduce adherence	May improve AD without systemic glucocorticoid exposure; ultraviolet B exposure can contribute to cutaneous vitamin D synthesis, although this is not a substitute for treating documented deficiency	No evidence supports phototherapy specifically for osteoporosis prevention. Its skeletal relevance is primarily indirect, through disease control and avoidance of systemic treatment [60,61].
Effective targeted systemic treatment as a therapeutic strategy	Sustained control of moderate-to-severe AD using an appropriately selected biologic or small-molecule agent	Unknown long-term skeletal effects because DXA, bone microarchitecture, and fractures are rarely prespecified trial outcomes	Reduction of inflammation, pruritus, sleep loss, inactivity, nutritional impairment, and systemic glucocorticoid exposure	Targeted therapy may reduce several mediators of bone fragility, but improvement in AD should not be equated with proven fracture prevention. Treatment selection should be individualized according to overall efficacy, safety, comorbidities, age, and baseline skeletal risk [58,59,60,61,62,63,64].

Abbreviations: AD, atopic dermatitis; BMD, bone mineral density; DXA, dual-energy X-ray absorptiometry; IL, interleukin; JAK, Janus kinase; OPG, osteoprotegerin.

## Data Availability

The data used to support the findings of this study are available from the corresponding author upon reasonable request.
